# Visible‐Light‐Initiated Palladium‐Catalyzed Cross‐coupling by PPh_3_ Uncaging from an Azobenzene Ruthenium–Arene Complex

**DOI:** 10.1002/chem.202200519

**Published:** 2022-06-10

**Authors:** Lou Rocard, Jérôme Hannedouche, Nicolas Bogliotti

**Affiliations:** ^1^ Université Paris-Saclay, ENS Paris-Saclay, CNRS Photophysique et Photochimie Supramoléculaires et Macromoléculaires 91190 Gif-sur-Yvette France; ^2^ Université Paris-Saclay, CNRS Institut de Chimie Moléculaire et des Matériaux d'Orsay (ICMMO) 91405 Orsay Cedex France

**Keywords:** arene ruthenium, azobenzene, photochemistry, Sonogashira cross-coupling, triphenylphosphine

## Abstract

Photo‐release of triphenylphosphine from a sulfonamide azobenzene ruthenium–arene complex was exploited to activate Pd^II^Cl_2_ into Pd^0^ catalyst, for the photo‐initiation of Sonogashira cross‐coupling. The transformation was initiated on demand – by using simple white LED strip lights – with a high temporal response and the ability to control reaction rate by changing the irradiation time. Various substrates were successfully applied to this photo‐initiated cross‐coupling, thus illustrating the wide functional‐group tolerance of our photo‐caged catalyst activator, without any need for sophisticated photochemistry apparatus.

## Graphical abstract

**Tuning by timing**: The photo‐initiation of Sonogashira cross‐coupling was achieved using a simple white LED combined with a triphenylphosphine ruthenium–arene complex bearing an azobenzene ligand. The strategy developed in this work involves the photo‐controlled release of metal catalyst activator (PPh_3_) through *Z*‐to‐*E* azobenzene isomerization, thus generating competent Pd^0^ active species, and allows fine tuning of reaction kinetics according to irradiation time.

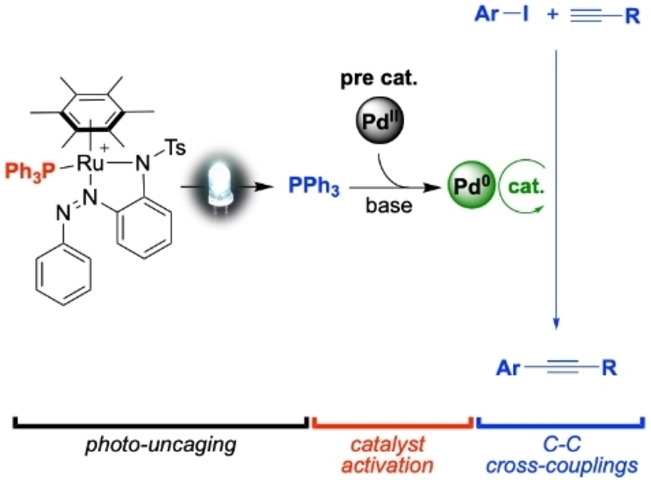

## Introduction

The field of photoswitchable catalysis has emerged over the last two decades from the idea of modulating the activity of a catalyst through the use of a non‐invasive external light stimulus, with high spatiotemporal response.^[1]^ In this respect, research efforts have focused on the incorporation of a light‐responsive actuator into the structure of a functional catalyst. In practice, however, maximizing the difference in reactivity between two forms of a photoswitchable catalyst remains a challenging and hardly a predictable issue. One reason lies in the inherent efficiency of the photoswitching process (notably photostationary state composition), which can be strongly influenced by the surrounding groups required for catalytic activity. Moreover, the modulation of reactivity originates from a variety of interconnected effects and subtle variations such as steric shielding, electronic modification, change in distance between reactive sites, or solubility and aggregation effects.^[1c]^

**Figure 1:**
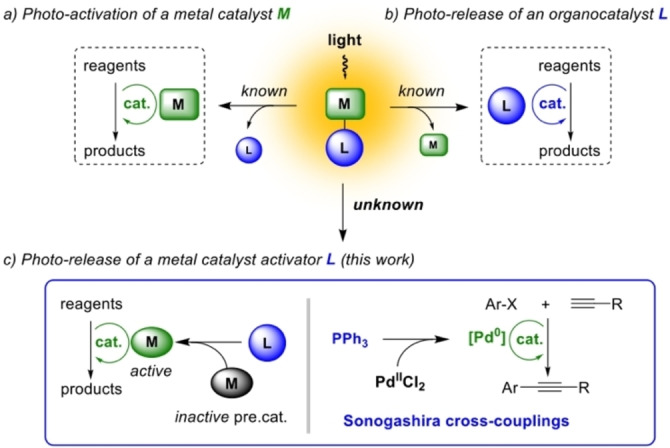
General paths for the induction of catalytic activity with light through “photo‐uncaging”.

The photo‐release of a chemical entity, often referred as “photo‐uncaging”, is conceptually and practically a much simpler process than photoswitching, providing unidirectional and irreversible generation of chemical entities. It has consequently been employed since the late 1970s as a tool for the control of biological processes and controlled release of bioactive substances.^[2]^ More recently, it has shown great promise for the development of efficient photoacid and photobase generators targeted towards polymeric materials and photoresist application.^[3]^ Such approach has also been successfully exploited for the generation of a vacant site at a metal center through ligand photo‐dissociation in various catalytic transformations such as alkene isomerization,^[4]^ and metathesis,^[5]^ [2+2+2] cycloadditions,^[6]^ allyl carbamate cleavage,^[7]^ or azide–thioalkyne cycloaddition^[8]^ (Figure 1 a). The strategy in which an organocatalyst is released from a photo‐active “cage” substrate has been also explored, though only few examples involving hexylamine (thiol‐Michael addition),^[9]^ proline (aldol, Michael and Mannich reaction),^[10]^ thiourea and squaramide (Michael addition),^[11]^ DMAP (acetylation of alcohols)^[12]^ and PPh_3_ (aza‐Morita−Baylis−Hillman reaction;^[13]^ Figure 1 b).^[14]^ To the best of our knowledge, the complementary approach involving photo‐release of a ligand that could in turn generate an active catalytic species from an appropriate precatalyst has not been reported to date (Figure 1 c). As a first example of this general strategy, we explored in this work, the ability of “photo‐uncaged” PPh_3_ to generate catalytically competent Pd^0^ species from Pd^II^ precatalysts and applied this process to the photo‐initiation of C(sp^2^)−C(sp) cross‐couplings, namely the Sonogashira reaction. As far as we know, no prior visible‐light‐activated palladium‐catalyzed coupling reaction has been achieved through photo‐release of the metal catalyst activator, as has with these PPh_3_.^[15]^

## Results and Discussion

**Scheme 1:**
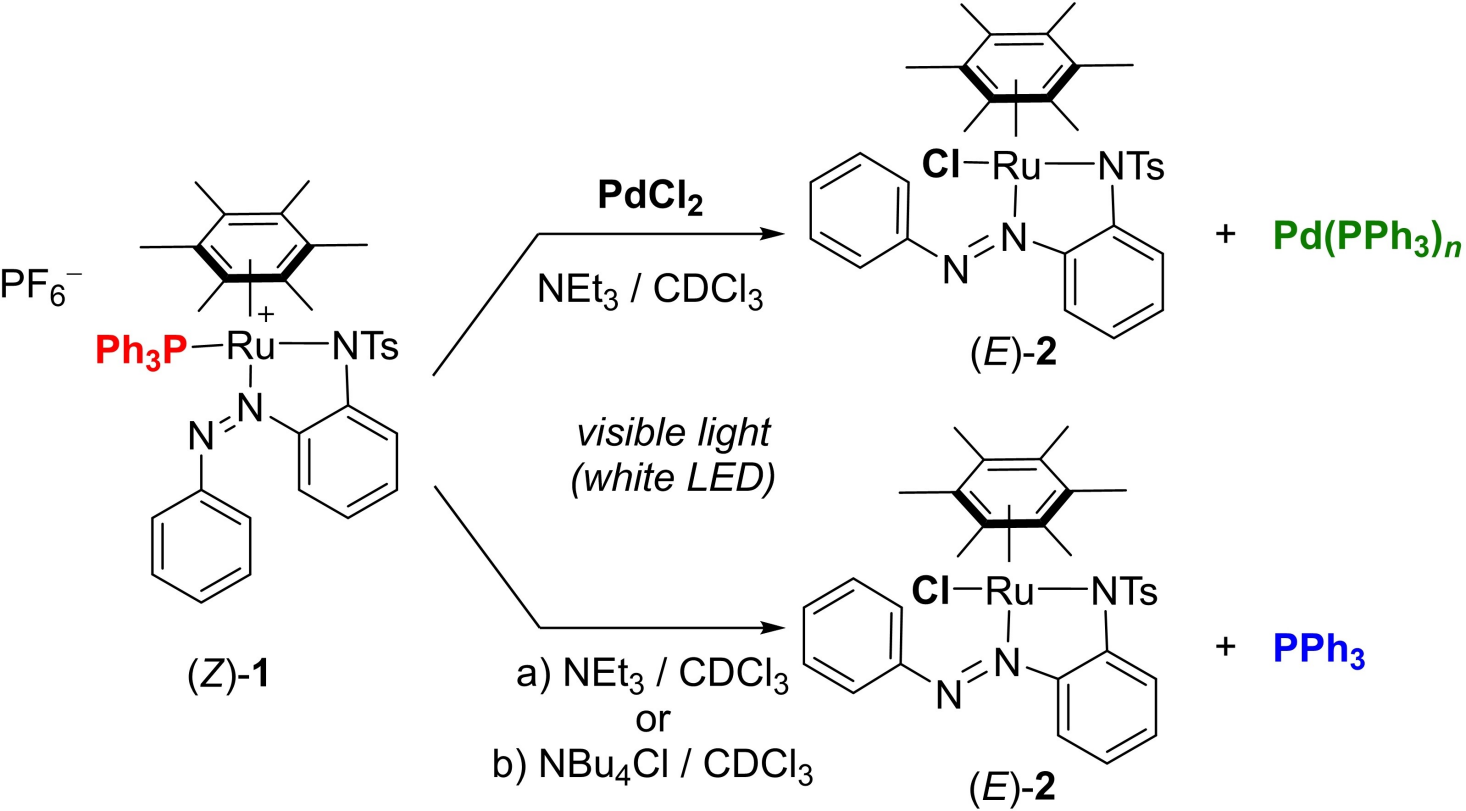
Photo‐uncaging of PPh_3_ from (*Z*)‐**1**. Photo‐induced reduction of PdCl_2_ (1 equiv.) into Pd(PPh_3_)_*n*_ with NEt_3_ (30 equiv; top) and photo‐release of PPh_3_ promoted by Cl^‐^ ions (bottom). a) NEt_3_ (70 equiv); b) NBu_4_Cl (5 equiv).

Upon visible light irradiation, ruthenium complex (*Z*)‐**1** ^[13]^ can release PPh_3_ and be converted into a new Ru^II^ species, through *Z→E* isomerization of the azobenzene core (Scheme 1). As demonstrated in our previous studies, the photo‐releasing efficiency is mostly affected by the nature of the surrounding medium.^[16]^ Noteworthily, in weakly coordinating solvents (such as dichloromethane, chloroform, or acetone), light irradiations led to a dynamic equilibrium consisting of about 50 % photo‐released PPh_3_ in mixture with (*Z*)‐**1** and H_2_O‐coordinated complex (Scheme S2 and Figure S7 in the Supporting Information), whereas quantitative phosphine release could be achieved in the presence of coordinating solvents (such as pyridine, DMSO, or acetonitrile).^[13]^

**Figure 2:**
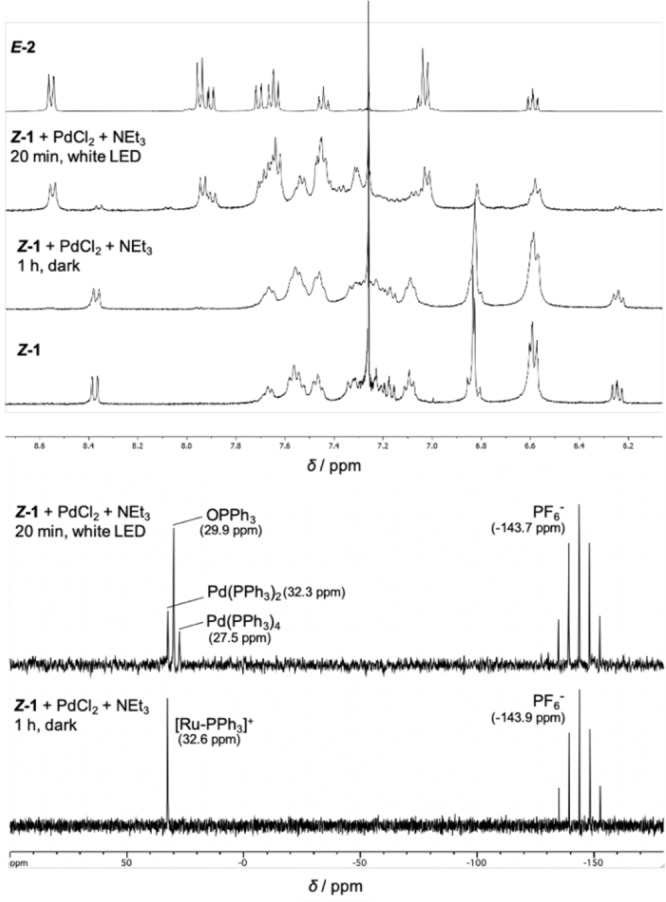
Evolution of ^1^H (top) and ^31^P (bottom) NMR spectra of (*Z*)‐**1** in CDCl_3_ (1 equiv.) along with PdCl_2_ (1 equiv.) and NEt_3_ (30 equiv.) after 1 h in the dark and after 20 min of light irradiation.

**Scheme 2:**
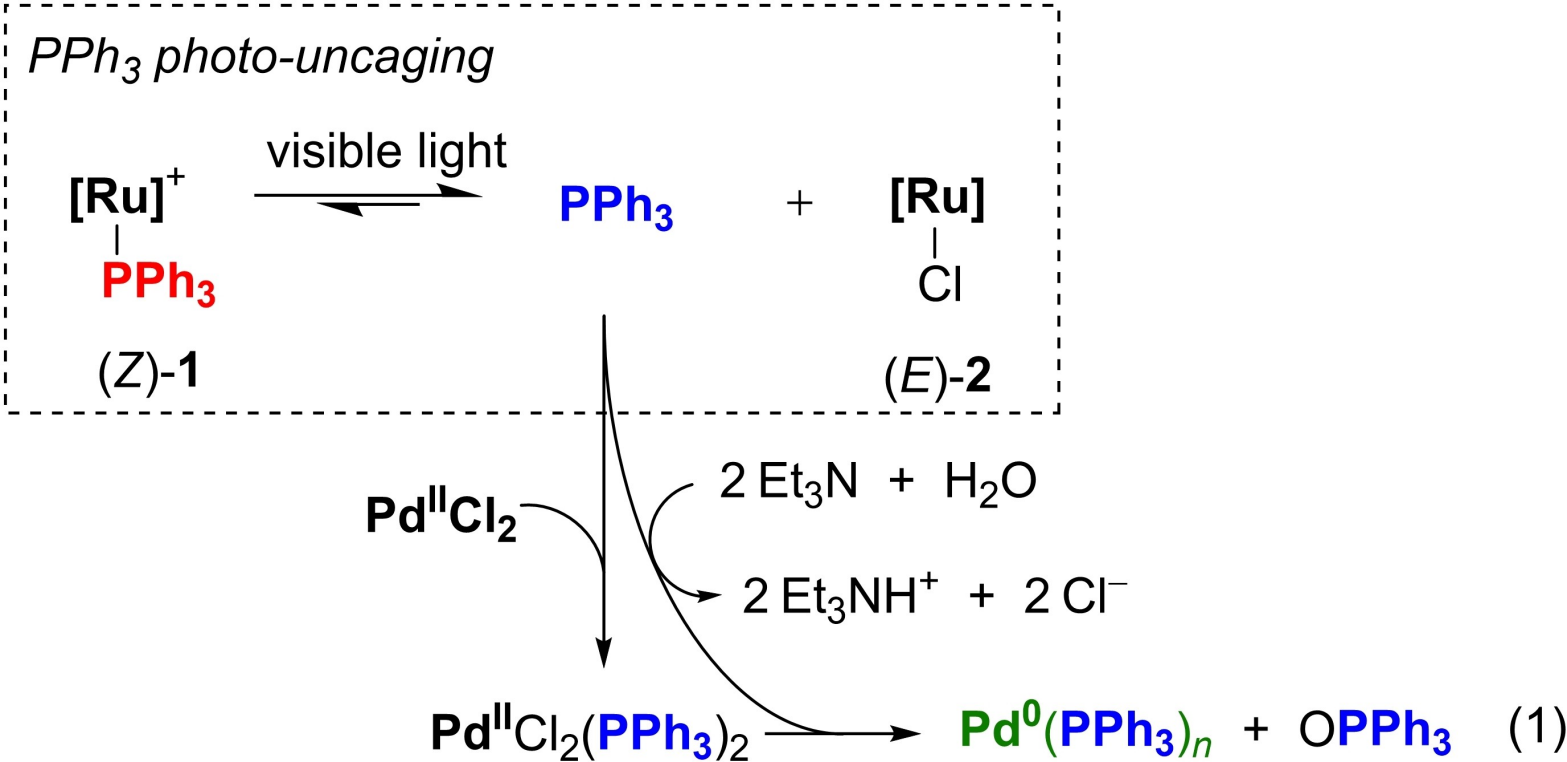
Plausible mechanism for the photo‐induced reduction of Pd^II^Cl_2_ to Pd^0^(PPh_3_)_*n*_.

In the aim of exploiting PPh_3_ photo‐release from (*Z*)‐**1** as a general platform for the initiation of a variety of chemical transformations with light, we decided to further investigate its reactivity with a focus on the reduction of Pd^II^Cl_2_ into active Pd^0^(PPh_3_)_*n*_ under basic conditions, to photo‐induce Pd‐catalyzed cross‐couplings.^[17]^ Preliminary studies revealed a good stability of (*Z*)‐**1** in the dark in a NEt_3_/CHCl_3_ ^[18]^ mixture, by comparison with other tested bases/solvents.^[19]^ Dispersing PdCl_2_ (1 equiv.) along with (*Z*)‐**1** (1 equiv.) and NEt_3_ (30 equiv.) in CDCl_3_, after 1 h in the dark, did not lead to any reaction, such as ligand exchange, and the characteristic signals of (*Z*)‐**1** remained unchanged according to ^1^H/^31^P NMR analyses (Figure 2). After 20 min of light irradiation using simple white LED (RGB) strip lights, ^1^H NMR showed quasi‐quantitative conversion of (*Z*)‐**1** with concomitant formation of (*E*)‐**2** (Scheme 1, top) and ^31^P NMR revealed new peaks: an intense one at 29.9 ppm and two smaller at 32.3 and 27.5 ppm, without sign of free PPh_3_ (−4.8 ppm) or PdCl_2_(PPh_3_)_2_ (24 ppm; Figure 2). According to experimental (Table S2) and literature data,^[20]^ the most intense signal (29.9 ppm) was ascribed to the formation of PPh_3_ oxide which might indicate the reduction of Pd^II^Cl_2_ into Pd^0^(PPh_3_)_*n*_ facilitated by the presence of traces of water^[21]^ in the medium (Scheme 2).^[17]^ If the signal at 32.3 ppm can be attributed to Pd(PPh_3_)_2_ along with traces of remaining (*Z*)‐**1**,^[22]^ the second lowest intense peak at 27.5 ppm could indicate the formation of Pd^0^(PPh_3_)_4_ complex according to the overall Equation (1) (Scheme 2).^[23]^

In comparison, irradiation for 15 min of (*Z*)‐**1** in CDCl_3_ in the presence of an excess of NEt_3_ but without PdCl_2_, also promoted the complete photo‐releasing with the concomitant formation of chloro complex (*E*)‐**2** (Scheme 1, bottom). This finding, supported by ^1^H NMR, UV‐Vis absorption studies (Figures S6–S8), suggested that chloride anions, that may originate from a solvent degradation under those conditions,^[24]^ also favored the efficient PPh_3_ uncaging.^[25]^ Indeed, upon light irradiations in the presence of an external source of chloride and without base, (*Z*)‐**1** was efficiently transformed into (*E*)‐**2** (Figure S9), ultimately confirming the fundamental cooperative role of chloride ions and light in the PPh_3_ uncaging (Scheme 1, bottom).

To assess the light‐driven ability of (*Z*)‐**1** to activate Pd^II^‐catalyzed cross‐couplings through PPh_3_ uncaging (and subsequent Pd^II^ reduction), the classical Sonogashira reaction was next targeted as typical procedures for this coupling involve NEt_3_ as the base along with Pd^II^ pre‐catalyst.^[26]^ We started our investigations by studying the cross‐coupling between an activated aryl iodide, 4‐iodoacetophenone, with trimethylsilyl (TMS) acetylene. Even though performing Sonogashira couplings in chlorinated solvents is uncommon, to our delight, the model reaction catalyzed by PdCl_2_/CuI in NEt_3_/CHCl_3_ can be efficiently turn on (100 % conv.) or off (<5 % conv.) according to the presence, or not, of PPh_3_ (4 mol%; Table 1, entries 1 and 2). When the reaction was performed under similar conditions with (*Z*)‐**1** (4 mol%) in the dark or under visible light irradiations, differences of reactivity were noticed with a complete conversion into cross‐coupled product under light compared with 45 % conversion in the dark (entry 3). This mild conversion obtained in the dark suggested a partial PPh_3_ releasing from (*Z*)‐**1** over the reaction, possibly favored by the presence of additional coordinating species (such as iodide ions). However, Cu‐free Sonogashira coupling appeared not to be suitable for this system (entry 4). Replacing (*Z*)‐**1** by (*E*)‐**2** did not promote the reaction (entry 5), thereby excluding a photosensitizing ability[^15a^, ^15b^, ^15d^, ^15f^, ^15g^] for our Ru^II^ complexes (or their decomposition into catalytically competent entities^[27]^) and highlighting the fundamental role of PPh_3_ ligand to generate and/or stabilize active Pd^0^ species.

**Table 1:** Investigations toward visible‐light‐initiated Sonogashira coupling.

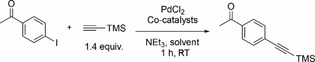					
	Solvent	Co‐catalysts	NEt_3_ [equiv.]	Yield [%]^[a]^	
				Irr. LED	Dark
1^[b]^	CHCl_3_	CuI	15	–	<5
2^[b]^	CHCl_3_	PPh_3_/CuI	15	–	100
3	CHCl_3_	(*Z*)‐**1**/CuI	15	100	45
4^[c]^	CHCl_3_	(*Z*)‐**1**	15	10	<5
5	CHCl_3_	(*E*)‐**2**/CuI	15	<5	–
6	CH_2_Cl_2_	(*Z*)‐**1**/CuI	15	100	95
7	CHCl_3_^[d]^	(*Z*)‐**1**/CuI	15	100	79
8	CHCl_3_^[e]^	(*Z*)‐**1**/CuI	15	93	55
9	CHCl_3_	(*Z*)‐**1**/CuI	2.5	100	30
**10**	**CHCl_3_**	**(*Z*)‐1/CuI**	**1.6**	**92**	**10**

Reaction scale: iodoaryl (0.057 mmol; 38 mM): PdCl_2_ (5 mol%) and co‐catalysts: CuI (6 mol%), (*Z*)‐**1**, PPh_3_ or (*E*)‐**2** (4 mol%). [a] NMR yields were determined from crude ^1^H NMR after workup based on the relative integrals product/remaining iodoaryl substrate. [b] Run under ambient light. [c] 4 h reaction time. [d] Neutralized through Al_2_O_3_ pad. [e] distilled over CaH_2_.

The effect of the solvent was next investigated. Switching to CH_2_Cl_2_, neutralized CHCl_3_ (passed through Al_2_O_3_ pad), or CHCl_3_ distilled over CaH_2_, reduced the “off effect” in the dark (entries 6–8). These experiments might suggest that the intrinsic acidity of CHCl_3_, used without further treatment, protected (*Z*)‐**1** from degradation under the cross‐coupling conditions employed herein.^[28]^ To our delight, decreasing the amount of the base from 15 to 2.5 or 1.6 equiv. significantly improved the on–off photo‐effect of the reaction. (entries 9 and 10). Indeed, a yield of 92 % yield was obtained when running the reaction upon irradiation with 1.6 equiv. of NEt_3_, in contrast to 10 % in the dark. This result demonstrated the potential of our photocaged system to efficiently turn on a Sonogashira coupling reaction upon visible light irradiation.

**Figure 3:**
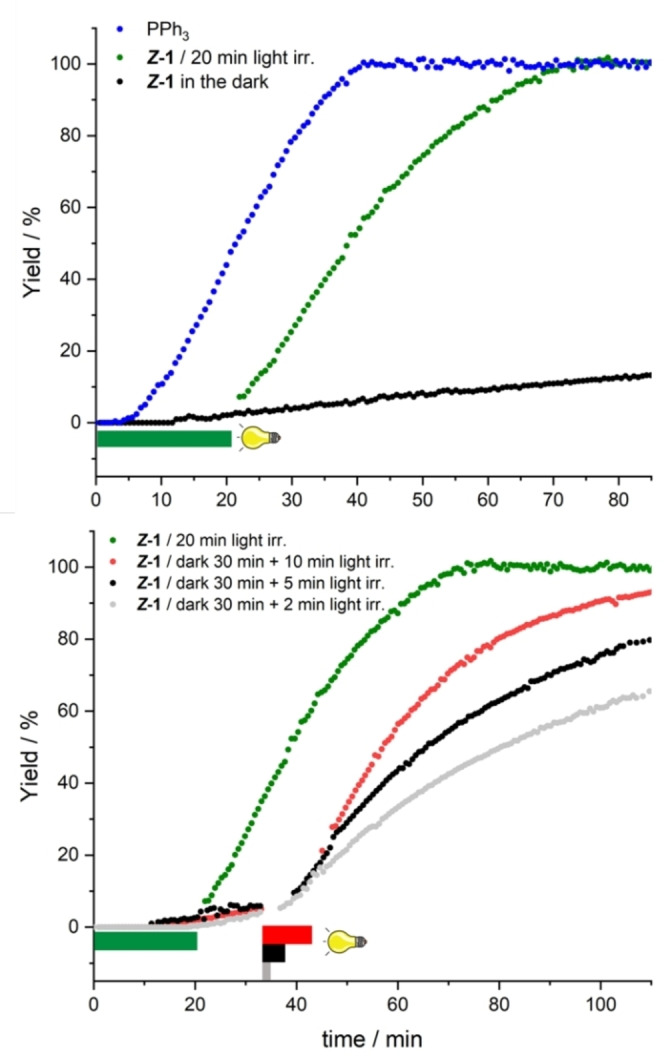
Yields of Sonogashira coupling between 4‐iodoacetophenone and TMS acetylene (conditions: Table 1, entry 10) determined by ^1^H NMR spectroscopy. Colored bars show length of visible light irradiation.

To gain more insights into the photo‐initiation of the coupling, conversion rates of our model reaction were investigated by ^1^H NMR over time, by directly performing the reaction into an NMR tube using PPh_3_ or (*Z*)‐**1** in the dark, or after light irradiations (Figure 3, top). The conversion rates appeared to be similar using PPh_3_ as ligand or (*Z*)‐**1** after 20 min of light exposure with a translational time of around 15 min, attributed to the irradiation period. When the reaction was carried out in the dark with (*Z*)‐**1**, no coupling occurred for 12 min and then, slowly took place. After 1 h, around 10 % of product was formed even though the degradation of (*Z*)‐**1** could not be detected by ^1^H NMR. On the other hand, while only negligible amount of product could be observed after 30 min in the dark, exposure to light for 10 min nicely initiated the cross‐coupling, to reach almost full conversion in about one additional hour (Figure 3, bottom). Interestingly, the rate of the coupling reaction could be finely tuned by changing the irradiation time for PPh_3_ photo‐releasing to 5 or 2 min. These experiments highlight the ability of our photocaged system to gradually respond to visible light exposure time on demand.

**Figure 4:**
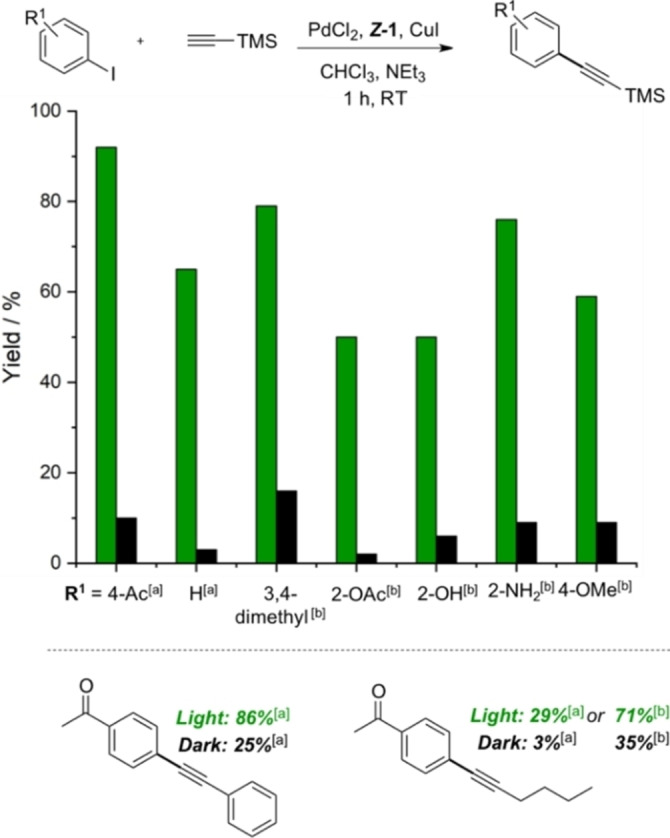
Light‐induced Sonogashira cross‐coupling between iodobenzene derivatives and acetylene derivatives by using (*Z*)‐**1**. Bar chart: variation of iodoaryl derivatives with TMS acetylene. Reaction conditions: PdCl_2_ (5 mol%), CuI (6 mol%), (*Z*)‐**1**, (4 mol%), NEt_3_ ([a] 1.6 or [b] 15 equiv). All yields were determined by ^1^H NMR spectroscopy.

To evaluate the efficiency of this light induced Sonogashira coupling on a broader substrate scope, the optimized conditions (Table 1, entry 10) were first applied to unsubstituted iodobenzene (R^1^=H) along with TMS‐acetylene, inducing a good on/off photo‐effect with 65 % yield upon irradiation versus traces of product when the reaction was performed in the dark (Figure 4; bar chart). To our delight, when the amount of base was increased to 15 equiv., the conversion was raised to 80 %. Same conditions were applied to other iodoaryl substrates, bearing deactivating substituents with electro‐donation or steric bulk.^[29]^ The reaction tolerated a wide range of functional groups on the arene ring, namely ketone, ester, alcohol, ether, amine. For all examples, turning the light on improved significantly the reaction reactivity as compared to the dark (Figure 4; bar chart). Aromatic and aliphatic acetylenes such as phenylacetylene or hex‐1‐yne were also suitable substrates for this reaction, yielding 86 and 71 % of product with light, compared with 25 and 35 % in the dark, respectively (Figure 4; bottom).

## Conclusion

Ruthenium complex (*Z*)‐**1**, acting as a light‐gated PPh_3_, was shown to promote the reduction of Pd^II^Cl_2_ into Pd^0^(PPh_3_)_*n*_ species upon irradiation with visible light from simple white LED strips under basic conditions in CHCl_3_. This process, which does not require sophisticated photochemistry apparatus, was applied to the photo‐initiation of Sonogashira cross‐couplings, whose rate could be finely tuned by changing the irradiation time. We anticipate that this concept could be transposed to other Pd^0^‐catalyzed transformations, or other metal catalytic systems, and open new horizons in the field of multicatalysis with the possibility of drastically changing the activity of a catalyst for relayed processes through external‐light stimulus.

## Conflict of interest

The authors declare no conflict of interest.

1

## Supporting information

As a service to our authors and readers, this journal provides supporting information supplied by the authors. Such materials are peer reviewed and may be re‐organized for online delivery, but are not copy‐edited or typeset. Technical support issues arising from supporting information (other than missing files) should be addressed to the authors.

Supporting Information

## Data Availability

The data that support the findings of this study are available in the supplementary material of this article.

## References

[1a] B. M. Neilson , C. W. Bielawski , ACS Catal. 2013, 3, 1874–1885;

[1b] R. Dorel , B. L. Feringa , Chem. Commun. 2019, 55, 6477–6486; 10.1039/c9cc01891c 31099809

[1c] S. P. Ihrig , F. Eisenreich , S. Hecht , Chem. Commun. 2019, 4290–4298; 10.1039/c9cc01431d 30924476

[1d] Z. Freixa , Catal. Sci. Technol. 2020, 10, 3122–3139;

[1e] F. Medici , N. Goual , V. Delattre , A. Voituriez , A. Marinetti , ChemCatChem 2020, 12, 5573–5589;

[1f] G. Sobczak , V. Sashuk , ChemCatChem 2021, 13, 506–513.

[2a] C. Brieke , F. Rohrbach , A. Gottschalk , G. Mayer , A. Heckel , Angew. Chem. Int. Ed. 2012, 51, 8446–8476; 10.1002/anie.201202134 22829531

[2b] A. Herrmann , Photochem. Photobiol. Sci. 2012, 11, 446–459; 22005713 10.1039/c1pp05231d

[2c] P. Klán , T. Šolomek , C. G. Bochet , A. Blanc , R. Givens , M. Rubina , V. Popik , A. Kostikov , J. Wirz , Chem. Rev. 2013, 113, 119–191; 23256727 10.1021/cr300177k PMC3557858

[2d] J. M. Silva , E. Silva , R. L. Reis , J. Controlled Release 2019, 298, 154–176; 10.1016/j.jconrel.2019.02.006 30742854

[2e] R. Weinstain , T. Slanina , D. Kand , P. Klán , Chem. Rev. 2020, 120, 13135–13272; 33125209 10.1021/acs.chemrev.0c00663 PMC7833475

[2f] L. Josa-Culleré , A. Llebaria , ChemPhotoChem 2021, 5, 296–314.

[3a] X. Sun , J. P. Gao , Z. Y. Wang , J. Am. Chem. Soc. 2008, 130, 8130–8131; 18528981 10.1021/ja802816g

[3b] R. S. Stoll , S. Hecht , Angew. Chem. Int. Ed. 2010, 49, 5054–5075; 10.1002/anie.201000146 20712033

[3c] C. J. Martin , G. Rapenne , T. Nakashima , T. Kawai , J. Photochem. Photobiol. C 2018, 34, 41–51;

[3d] N. Zivic , P. K. Kuroishi , F. Dumur , D. Gigmes , A. P. Dove , H. Sardon , Angew. Chem. Int. Ed. 2019, 58, 10410–10422; 10.1002/anie.201810118 30575230

[3e] H. Lai , J. Zhang , F. Xing , P. Xiao , Chem. Soc. Rev. 2020, 49, 1867–1886. 32101186 10.1039/c9cs00731h

[4] M. A. Schroeder , M. S. Wrighton , J. Am. Chem. Soc. 1976, 98, 551–558.

[5a] M. Picquet , C. Bruneau , P. H. Dixneuf , Chem. Commun. 1998, 2249–2250;

[5b] L. Delaude , A. Demonceau , A. F. Noels , Chem. Commun. 2001, 986–987;

[5c] D. Wang , K. Wurst , W. Knolle , U. Decker , L. Prager , S. Naumov , M. R. Buchmeiser , Angew. Chem. Int. Ed. 2008, 47, 3267–3270; 10.1002/anie.200705220 18338361

[5d] A. Ben-Asuly , A. Aharoni , C. E. Diesendruck , Y. Vidavsky , I. Goldberg , B. F. Straub , N. G. Lemcoff , Organometallics 2009, 28, 4652–4655;

[5e] C. Theunissen , M. A. Ashley , T. Rovis , J. Am. Chem. Soc. 2019, 141, 6791–6796. 31010292 10.1021/jacs.8b13663 PMC7097883

[6] K. P. C. Vollhardt , Angew. Chem. Int. Ed. 1984, 23, 539–556;

[7] P. K. Sasmal , S. Carregal-Romero , W. J. Parak , E. Meggers , Organometallics 2012, 31, 5968–5970.

[8] A. Gutiérrez-González , P. Destito , J. R. Couceiro , C. Pérez-González , F. López , J. L. Mascareñas , Angew. Chem. Int. Ed. 2021, 60, 16059–16066. 10.1002/anie.202103645 PMC9545742 33971072

[9] W. Xi , M. Krieger , C. J. Kloxin , C. N. Bowman , Chem. Commun. 2013, 49, 4504–4506. 10.1039/c3cc41123k 23572056

[10a] C. Maity , F. Trausel , R. Eelkema , Chem. Sci. 2018, 9, 5999–6005; 30079215 10.1039/c8sc02019a PMC6050528

[10b] C. Guruge , S. Y. Rfaish , C. Byrd , S. Yang , A. K. Starrett , E. Guisbert , N. Nesnas , J. Org. Chem. 2019, 84, 5236–5244. 30908906 10.1021/acs.joc.9b00220 PMC6510250

[11] K. Grill , H. Dube , J. Am. Chem. Soc. 2020, 142, 19300–19307. 33112151 10.1021/jacs.0c09519

[12] J. A. González-Delgado , M. A. Romero , F. Boscá , J. F. Arteaga , U. Pischel , Chem. Eur. J. 2020, 26, 14229–14235. 32449554 10.1002/chem.202001893

[13] C. Deo , N. Bogliotti , P. Retailleau , J. Xie , Organometallics 2016, 35, 2694–2700.

[14] An original all-organic related approach, in which a condensed aromatic carbocation exhibiting Lewis acid character – generated from photochromic terarylene – initiate a catalytic Mukaiyama-aldol reaction, was also reported: R. Mizutsu , R. Asato , C. J. Martin , M. Yamada , Y. Nishikawa , S. Katao , M. Yamada , T. Nakashima , T. Kawai , J. Am. Chem. Soc. 2019, 141, 20043–20047. 31814390 10.1021/jacs.9b11821

[15] Other strategies for light-controlled Pd-catalyzed reactions, through metallaphotocatalysis, photoswitchable phosphines or photoswitchable palladium complexes:

[15a] M. Osawa , H. Nagai , M. Akita , Dalton Trans. 2007, 827–829; 17297509 10.1039/b618007h

[15b] D. Kalyani , K. B. McMurtrey , S. R. Neufeldt , M. S. Sanford , J. Am. Chem. Soc. 2011, 133, 18566–18569; 22047138 10.1021/ja208068w PMC3222896

[15c] Z. S. Kean , S. Akbulatov , Y. Tian , R. A. Widenhoefer , R. Boulatov , S. L. Craig , Angew. Chem. Int. Ed. 2014, 53, 14508–14511; 10.1002/anie.201407494 25359436

[15d] K. Mori , M. Kawashima , H. Yamashita , Chem. Commun. 2014, 50, 14501–14503; 10.1039/c4cc03682d 24943224

[15e] D. Zhao , T. M. Neubauer , B. L. Feringa , Nat. Commun. 2015, 6, 6652; 25806856 10.1038/ncomms7652 PMC4389239

[15f] H. Zhang , X. Huang , Adv. Synth. Catal. 2016, 358, 3736–3742;

[15g] S. S. Babu , M. Shahid , P. Gopinath , Chem. Commun. 2020, 56, 5985–5988; 10.1039/d0cc01443e 32347860

[15h] M. Shee , N. D. P. Singh , Catal. Sci. Technol. 2021, 11, 742–767;

[15i] A. Kunfi , I. Jablonkai , T. Gazdag , P. J. Mayer , P. P. Kalapos , K. Németh , T. Holczbauer , G. London , RSC Adv. 2021, 11, 23419–23429. 35479800 10.1039/d1ra03838a PMC9036612

[16] Such behaviour was previously observed upon monochromatic irradiations at 405 nm (see ref. [13]).

[17a] V. V. Grushin , H. Alper , Organometallics 1993, 12, 1890–1901;

[17b] C. Amatore , L. El Kaïm , L. Grimaud , A. Jutand , A. Meignié , G. Romanov , Eur. J. Org. Chem. 2014, 2014, 4709–4713.

[18] Solvent directly used from the bottle without any prior treatment.

[19] For example, with an inorganic base such as K_2_CO_3_ (10 equiv.) in a THF/H_2_O mixture in the dark, decomposition of (*Z*)-**1** occurred within 10 min. In the presence of a large excess of triethylamine (50–80 equiv.) in the dark (*Z*)-**1** revealed a good stability in CH_2_Cl_2_ or CHCl_3_ in contrast to more polar solvents such as THF or [D_6_]acetone (see Section 3 in the Supporting Information for details).

[20] M. Gazvoda , M. Virant , B. Pinter , J. Košmrlj , Nat. Commun. 2018, 9, 4814. 30446654 10.1038/s41467-018-07081-5 PMC6240041

[21] According to Karl Fisher titrations, (untreated) CDCl_3_ and CHCl_3_ contained 65 and 200 ppm of water, respectively.

[22] The contribution of remaining traces of (*Z*)-**1** to this signal is weak, as shown by the ^1^H NMR spectrum.

[23] Pd^0^(PPh_3_)_4_ 27.7 ppm, see ref. [20] and the Supporting Information.

[24a] A. T. Chapman , J. Am. Chem. Soc. 1935, 57, 419–422;

[24b] J. Hine , J. Am. Chem. Soc. 1950, 72, 2438–2445.

[25] The ^1^H and ^31^P NMR spectra (Figure S8) also reveal the additional presence of OPPh_3_ in the reaction medium.

[26a] R. Chinchilla , C. Nájera , Chem. Soc. Rev. 2011, 40, 5084–5121; 21655588 10.1039/c1cs15071e

[26b] I. Kanwal , A. Mujahid , N. Rasool , K. Rizwan , A. Malik , G. Ahmad , S. A. A. Shah , U. Rashid , N. M. Nasir , Catalysts 2020, 10, 443.

[27] S. Park , M. Kim , D. H. Koo , S. Chang , Adv. Synth. Catal. 2004, 346, 1638–1640.

[28] We speculate that partial degradation of (*Z*)-**1** is promoted by hydroxide ions; and so, reducing their concentration limits this side-effect.

[29] M. Schilz , H. Plenio , J. Org. Chem. 2012, 77, 2798–2807. 22390837 10.1021/jo202644g

