# Supplementary material for: Visible‐Light‐Initiated Palladium‐Catalyzed Cross‐coupling by PPh3 Uncaging from an Azobenzene Ruthenium–Arene Complex
Source: Chemistry. 2022 Jun 10;28(41):e202200519. doi: 10.1002/chem.202200519 (PMC9400985; doi:10.1002/chem.202200519)
Supplement: Supplementary file 1 — Supporting Information [file CHEM-28-0-s001.pdf]

Chemistry–A European Journal

Supporting Information

Visible-Light-Initiated Palladium-Catalyzed Cross-coupling by PPh_3 Uncaging from an Azobenzene Ruthenium–Arene Complex

Lou Rocard, Jérôme Hannedouche,* and Nicolas Bogliotti*

Supporting Information

Table of Contents

1. Materials and Methods 2
2. Synthesis and characterization of Ruthenium complexes 3
3. Stability of ***Z*-1** toward base in the dark 4
4. Photoreleasing of PPh_3 from ***Z*-1** 7
5. Photo-induced reduction of $PdCl_2$ 12
6. Sonogashira cross-couplings 15
7. Appendix 17
8. References 20

1. Materials and Methods

Chemicals and solvents were purchased from Sigma Aldrich, Acros Organics, Fisher Scientific, Alfa Aesar, Fluorochem, and were used without purification. Deuterated solvents were purchased from Eurisotop. The amount of water contained in commercial $CHCl_3$ and $CDCl_3$ was estimated around 200 ppm and 65 ppm respectively, by Karl Fisher titrations (C20SX Coulometric KF Titrator). Thin Layer Chromatography (TLC) was conducted on pre-coated aluminum sheets with 0.20 mm MerckAlugram SIL G/UV254 with fluorescent indicator UV254. Column chromatography was carried out on CombiFlash-Rf with UV detection (two channels). UV-Vis absorptions were recorded on a Cary 5000 UV-Vis spectrophotometer from Agilent Technologies using quartz cell (pathlength of 1 cm). Emission spectrum of the light source was recorded on a spectrofluorometer Fluorolog FL3-221 from Horiba Jobin-Yvon by hiding the light source of the instrument. Nuclear magnetic resonance (NMR) ^{1}H and ^{31}P spectra were obtained on a Jeol ECS-400 spectrometer (400 MHz for ^{1}H and 162 MHz for ^{31}P) or 400 MHz Bruker spectrometer. Chemical shifts were reported in ppm according to tetramethylsilane using the solvent residual signal as an internal reference ($CDCl_3$: δ_H= 7.26 ppm). Coupling constants (*J*) were given in Hz. Resonance multiplicity was described as s (singlet), d (doublet), t (triplet), dd (doublet of doublets), m (multiplet) and hept. (heptuplet). Phosphorous spectra were acquired with a complete decoupling for the proton.

Photoinduced reactions were performed using 1) continuous irradiations with Hg/Xe lamp (Hamamatsu, LC6 Lightningcure, P=40 mW/cm^2 @405 nm) equipped with a narrow band interference filter of appropriate wavelength (Semrock FF01-406/15-25 for λ_{irr}= 405 nm) for the synthesis of ***Z*-1**[1] ; or 2) an homemade reactor with cold white LED (RGB) strip lights

(SMD 5050 LED strip; P=1.6 mW/cm^2 @500 nm measured at the center, where the reaction vessel was placed) enrolled in a crystallizer covered by aluminum foal (for photoreleasing PPh_3 from ***Z*-1**). The irradiation power was measured using a photodiode from Ophir (PD300-UV).

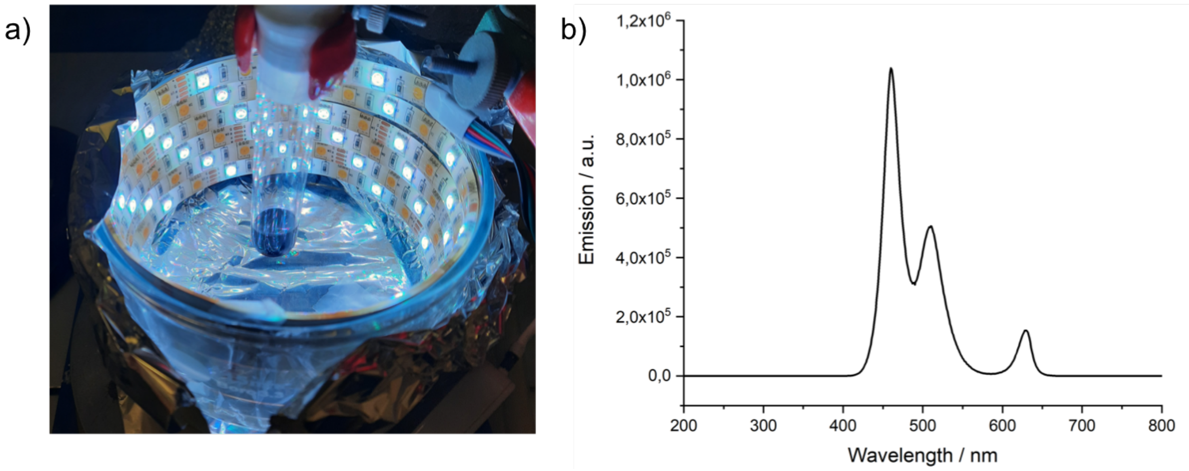

Figure S1. a) Picture of the homemade reactor with cold white LED (RGB) strip lights with glass tube placed at the center. b) Emission spectrum of the LED strip lights.

2. Synthesis and characterization of Ruthenium complexes

For the synthesis of tosylamide azobenzene Ruthenium-hmbz complex ***Z*-1**, our previously reported procedure was slightly modified.[1]

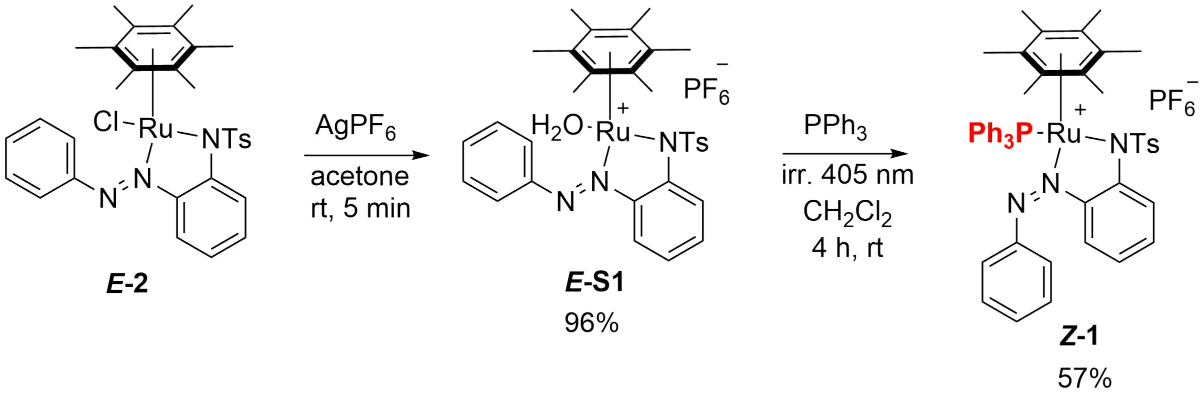

Scheme S1. Synthesis of photoswitchable Ruthenium complex ***Z*-1**.

***E*-2** was synthesized and characterized in accordance with previous report.[2] ^{1}H NMR (400 MHz, $CDCl_3$): δ 8.56 (d, *J* = 7.8 Hz, 2H), 7.95 (d, *J* = 8.7 Hz, 2H), 7.91 (d, *J* = 8.2 Hz, 1H), 7.71 (d, *J* = 8.7 Hz, 1H), 7.67-7.62 (m, 2H), 7.46-7.43 (m, 1H), 7.07-7.01 (m, 3H), 6.61-6.57 (m, 1H), 2.22 (s, 3H), 1.81 (s, 18H).

***E*-S1.** To a solution of ***E*-2** (1 g; 1.54 mmol) in acetone (120 mL) under Argon atmosphere, freshly opened $AgPF_6$ (584 mg; 2.51 mmol) was added and the mixture was stirred at rt for 5 min. The solution was filtered off and the filtrate was concentrated under reduced pressure. The crude residue was

precipitated in $CHCl_3$, filtered, washed with $CHCl_3$ and Et_2O to afford ***E*-S1** as a brown solid. (1.15 g; 96% yield). Characterizations in acetone-d_6 were in accordance with previous report,[1] and ^{1}H NMR (400 MHz) recorded in $CDCl_3$ is depicted in Figure S14 as reference.

***Z*-1.** A solution of ***E*-S1** (200 mg; 0.26 mmol) and PPh_3 (675 mg; 2.57 mmol) in CH_2Cl_2 (200 mL) was stirred upon irradiations at 405 nm for 4 h. The solution was concentrated under reduced pressure and the residue was dissolved in a minimum amount of CH_2Cl_2 and precipitated in toluene. The precipitate was filtered and washed with toluene and purified through SiO_2 column chromatography (gradient CH_2Cl_2 to CH_2Cl_2/acetone 95:5) to afford ***Z*-1** as a dark red solid (151 mg; 57%). Characterizations in acetone-d_6 were in accordance with previous report.[1] ^{1}H NMR (400 MHz, $CDCl_3$): δ 8.38 (d, J = 8.9 Hz, 1H), 7.69-7.65 (m, 2H), 7.58-7.52 (m, 4H), 7.46-7.44 (m, 2H), 7.34-7.15 (m, 6H), 7.09 (t, J = 7.6 Hz, 2H), 6.85-6.81 (m, 4H), 6.60-6.57 (m, 6H), 6.25 (t, J = 8.3 Hz, 1H), 2.24 (s, 3H), 1.91 (s, 18H); ^{31}P NMR (162 MHz, $CDCl_3$): δ 32.6 (s), -143.9 (hept., J = 907 Hz).

Solutions of ***E*-2**, ***E*-S1** and ***Z*-1** in $CHCl_3$ were analyzed by steady-state absorption UV-Vis spectroscopy (Figure S2, Table S1).

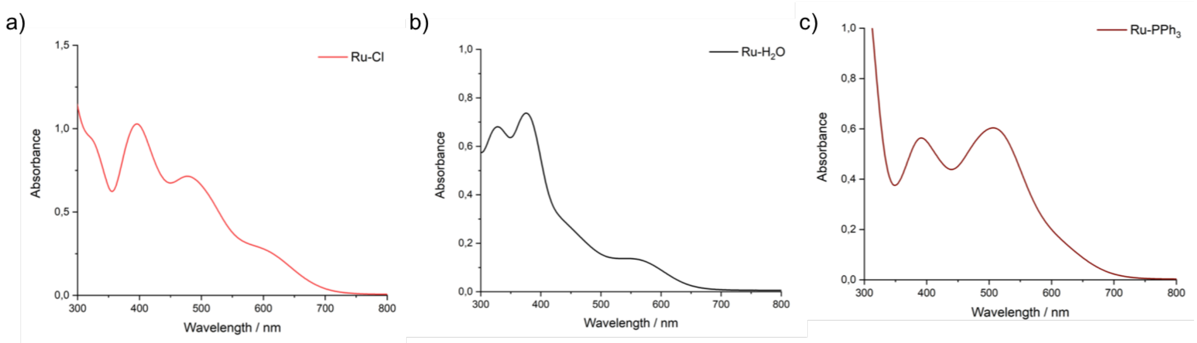

Figure S2. UV-Vis absorption spectra in $CHCl_3$ of a) ***E*-2** (1.3×10^{-4} M) with ; b) ***E*-S1** (9.8×10^{-5} M); c) ***Z*-1** (9.8×10^{-5} M).

Table S1. Optical properties of the complexes in $CHCl_3$. Extinction coefficient not determined for ***E*-S1** due to its low solubility.

Compounds	λ_{max} / nm	ε (λ_{max}) / $M^{-1}cm^{-1}$
***E*-2**	396, 480	$\varepsilon_{396} = 7.5 \times 10^3$
***E*-S1**	329, 376, 564	nd
***Z*-1**	393, 506	$\varepsilon_{393} = 5.8 \times 10^3$<br>$\varepsilon_{506} = 6.2 \times 10^3$

3. Stability of *Z*-1 toward base in the dark

Preliminary studies were conducted to investigate its stability in the dark under basic conditions. With an inorganic base (such as 10 equiv. of K_2CO_3 in THF/H_2O), decomposition of ***Z*-1** occurred rapidly

(within 10 min). In the presence of an excess of triethylamine (50 equiv. of NEt_3), the stability of the complex strongly depends on the solvents. In polar organic solvents (*i.e.* THF, CH_3CN), progressive degradation of ***Z*-1** was observed by TLC over 2 h and confirmed by ^1H NMR in acetone-d6 in only 50 min (Figure S3). The degradation was accelerated by adding water (occurred in less than 10 min).

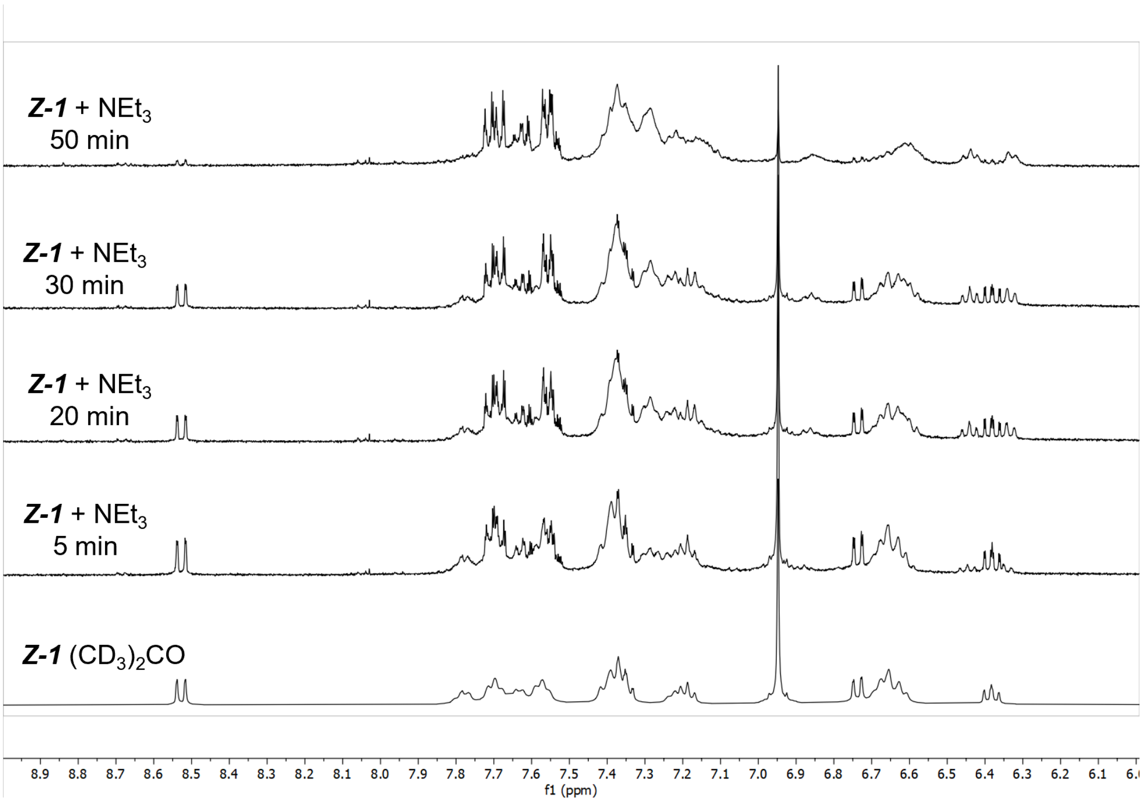

Figure S3. Evolution of ^1H NMR (400 MHz) spectrum of ***Z*-1** (8 mM; 1 equiv.) in acetone-d_6 with NEt_3 (17 equiv.) showing the complete degradation of ***Z*-1** over 50 min in the dark.

In non-coordinating chlorinated solvents (CD_2Cl_2 or $CDCl_3$), ***Z*-1** appeared to be much more stable in the presence of an excess of triethylamine in the dark. This was confirmed by ^1H NMR (2.8 mM in $CDCl_3$ or CD_2Cl_2 with 70 equiv. of NEt_3) (Figure S4) and UV-Vis absorption spectroscopy (9.8×10^{-5} M in $CHCl_3$ with 50 equiv. of NEt_3), which reveal unchanged spectra 1 h after the NEt_3 addition (Figure S5).

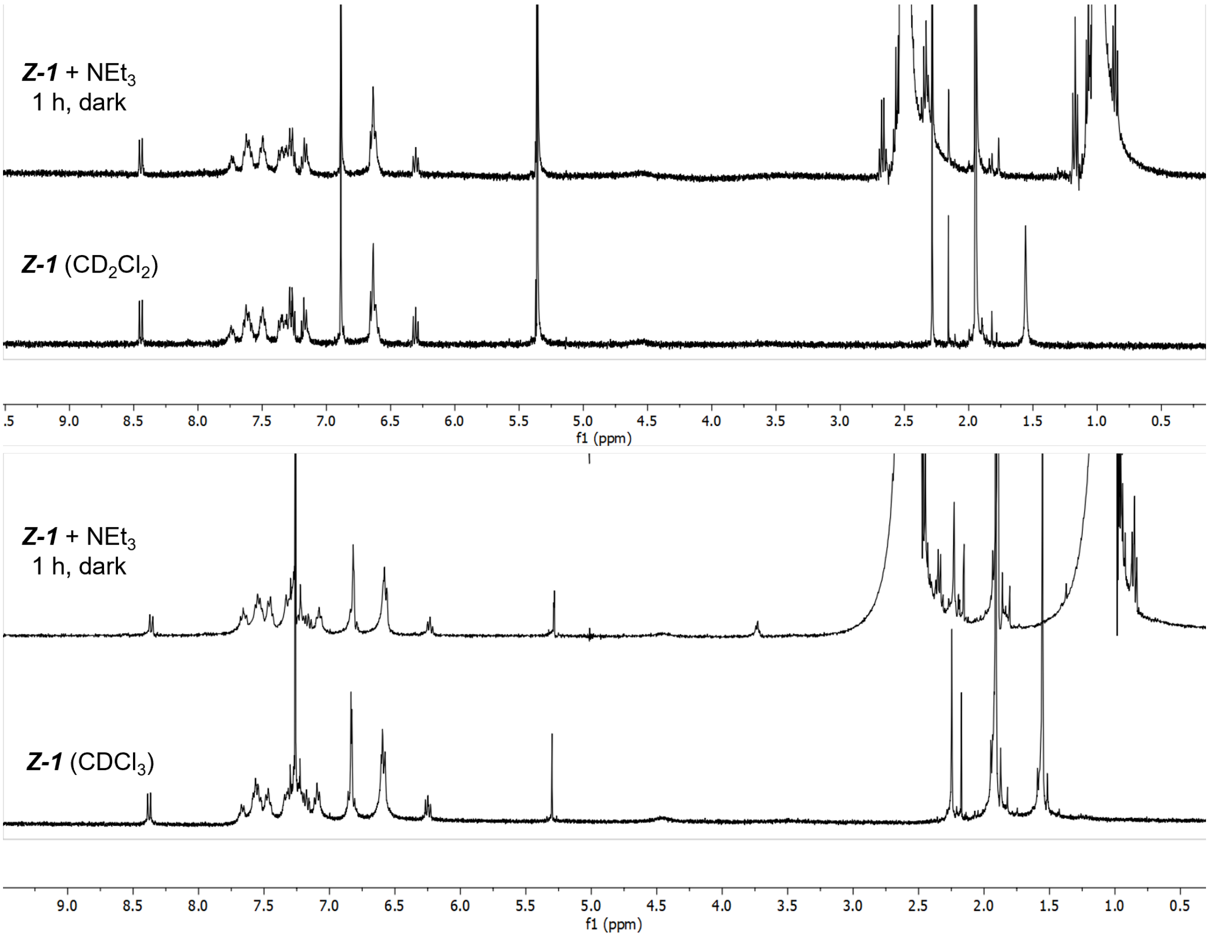

Figure S4. Evolution of ^{1}H NMR (400 MHz) spectrum of ***Z*-1** (1 equiv.; 3 or 2.5 mM) in top: CD_2Cl_2 or bottom: $CDCl_3$ with NEt_3 (70 equiv.) showing stable ***Z*-1** over 1 h in the dark.

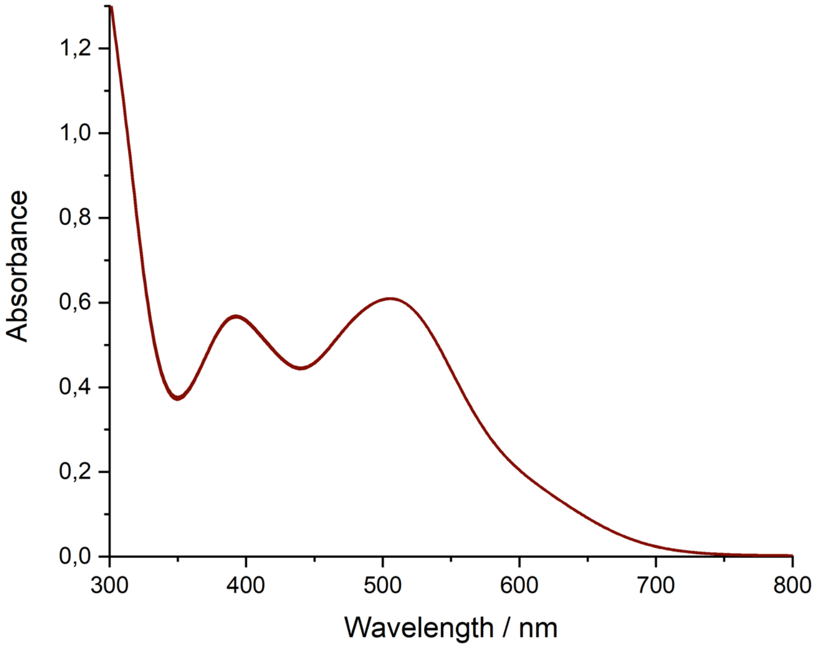

Figure S5. Superimposed UV-Vis absorption of ***Z*-1** in $CHCl_3$ (9.8×10^{-5} M) before and after the addition of NEt_3 (50 equiv.) for 1 h in the dark.

4. Photoreleasing of PPh_3 from *Z*-1

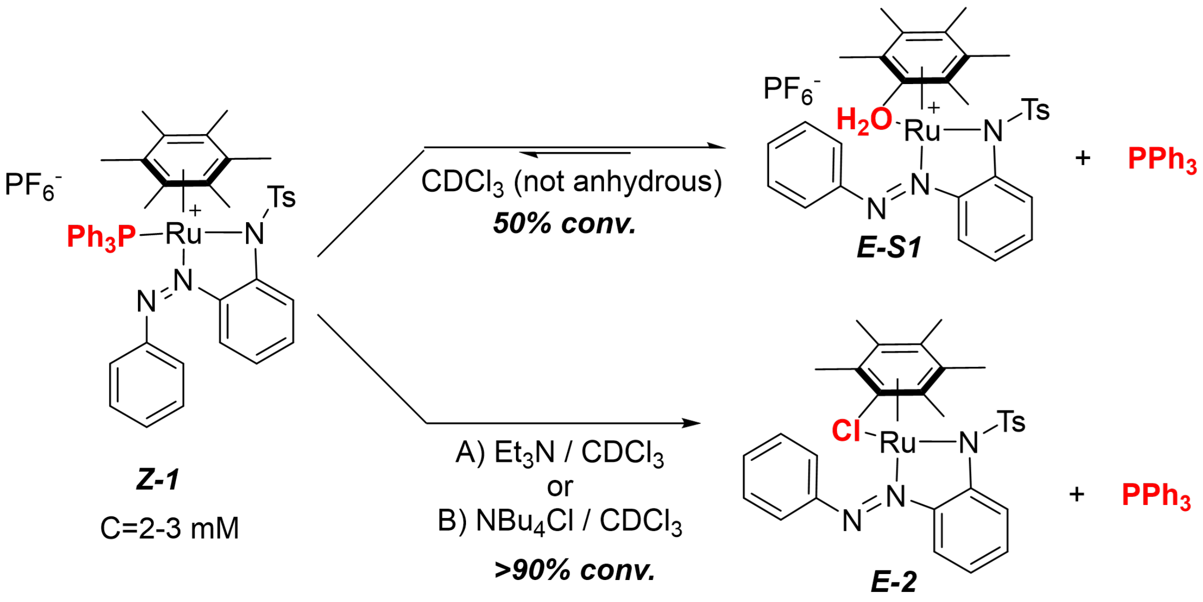

Scheme S2. Dual behaviour of triphenylphosphine photo-uncaging from ***Z*-1** upon 15 min of white light (LED) irradiations. Top: $CDCl_3$ (***Z*-1**: 2.5 mM). Bottom: Method A) NEt_3 (70 equiv.) / $CDCl_3$ (***Z*-1**: 2.5 mM); method B) NBu_4Cl (5 equiv.) / $CDCl_3$ (***Z*-1**: 2.8 mM).

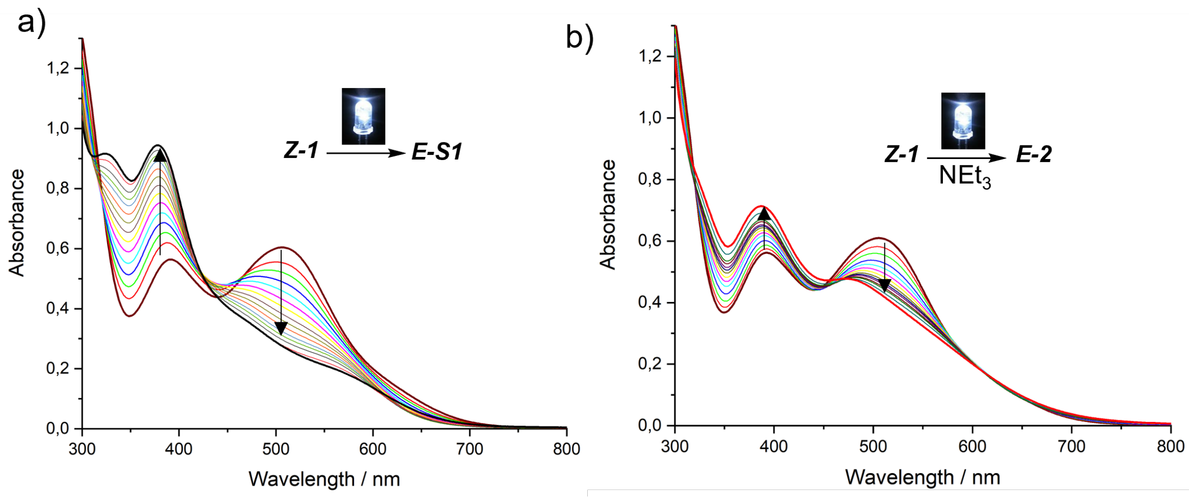

Figure S6. Evolution of the UV-Vis absorption of ***Z*-1** in $CHCl_3$ (C=0.98 µM) after light irradiations a) without NEt_3; b) with NEt_3 (50 equiv.). The 6 first spectra were recorded each 10s of light irr.; then 2 each 15s; then 2 each 20s; then 2 each 30s; then 1 after additional 60s; the final one after additional 120s (total irradiation time = 6min10s). See Figure S2 for reference spectra.

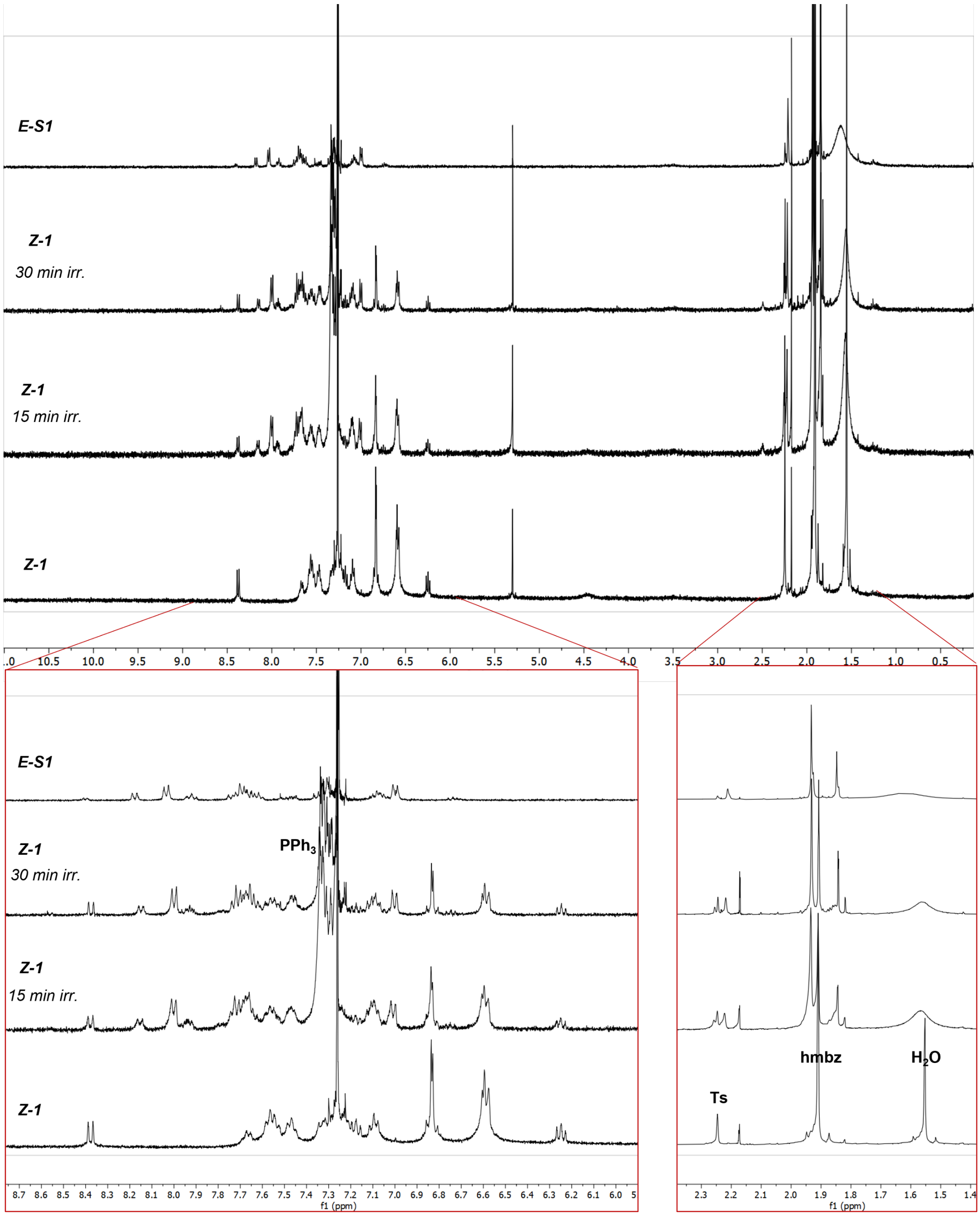

Figure S7. Evolution of ^{1}H NMR (400 MHz) spectrum of ***Z*-1** (2.5 mM) in $CDCl_3$ upon white light irradiations (15 and 30 min) affording a mixture of ***Z*-1** (*ca.* 50%) and ***E*-S1** / PPh_3 (*ca.* 50%) (compared with ^{1}H NMR spectrum of ***E*-S1**). Inset: Zoom of the aromatic and the aliphatic regions.

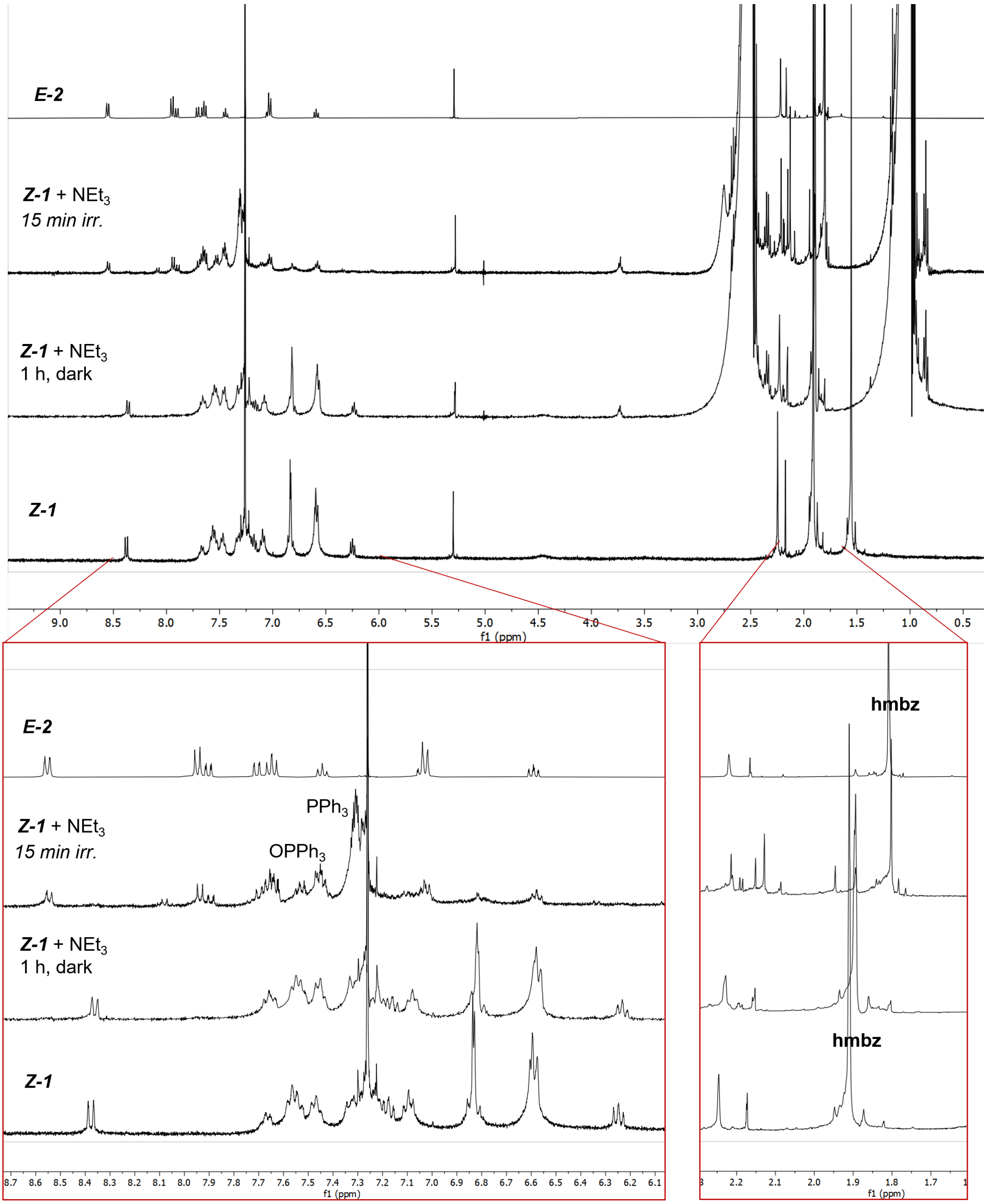
E-2
Z-1 + NEt3
15 min irr.
Z-1 + NEt3
1 h, dark
Z-1
f1 (ppm)
E-2
PPh3
Z-1 + NEt3
15 min irr.
OPPh3
Z-1 + NEt3
1 h, dark
Z-1
f1 (ppm)
hmbz
hmbz
f1 (ppm)

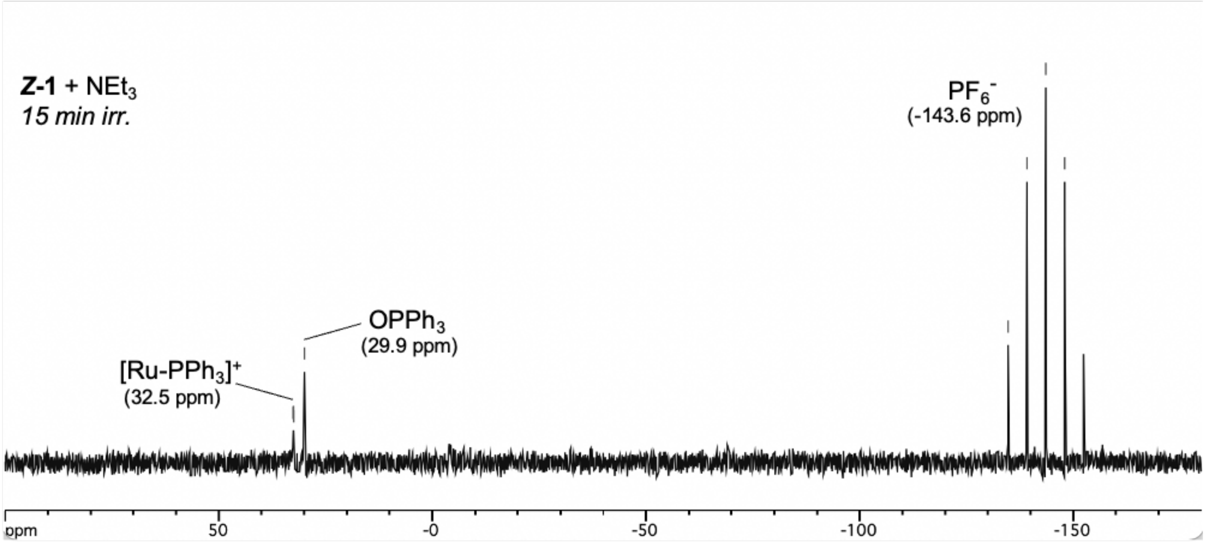

Figure S8. (Top) Evolution of ^{1}H NMR (400 MHz) spectrum of ***Z*-1** (2.5 mM) in $CDCl_3$ after NEt_3 (70 equiv.) addition for 1 h in the dark; and 15 min upon white light irradiations (compared with ^{1}H NMR spectrum of ***E-2***). Inset: Zoom of the aromatic and the aliphatic regions. (Bottom) ^{31}P NMR (162 MHz) spectrum of ***Z*-1** (2.5 mM) in $CDCl_3$ after NEt_3 (70 equiv.) addition and 15 min white light irradiation. *NB: In contrast to the ^{1}H NMR spectrum, free PPh_3 signals are not clearly visible by ^{31}P NMR.*

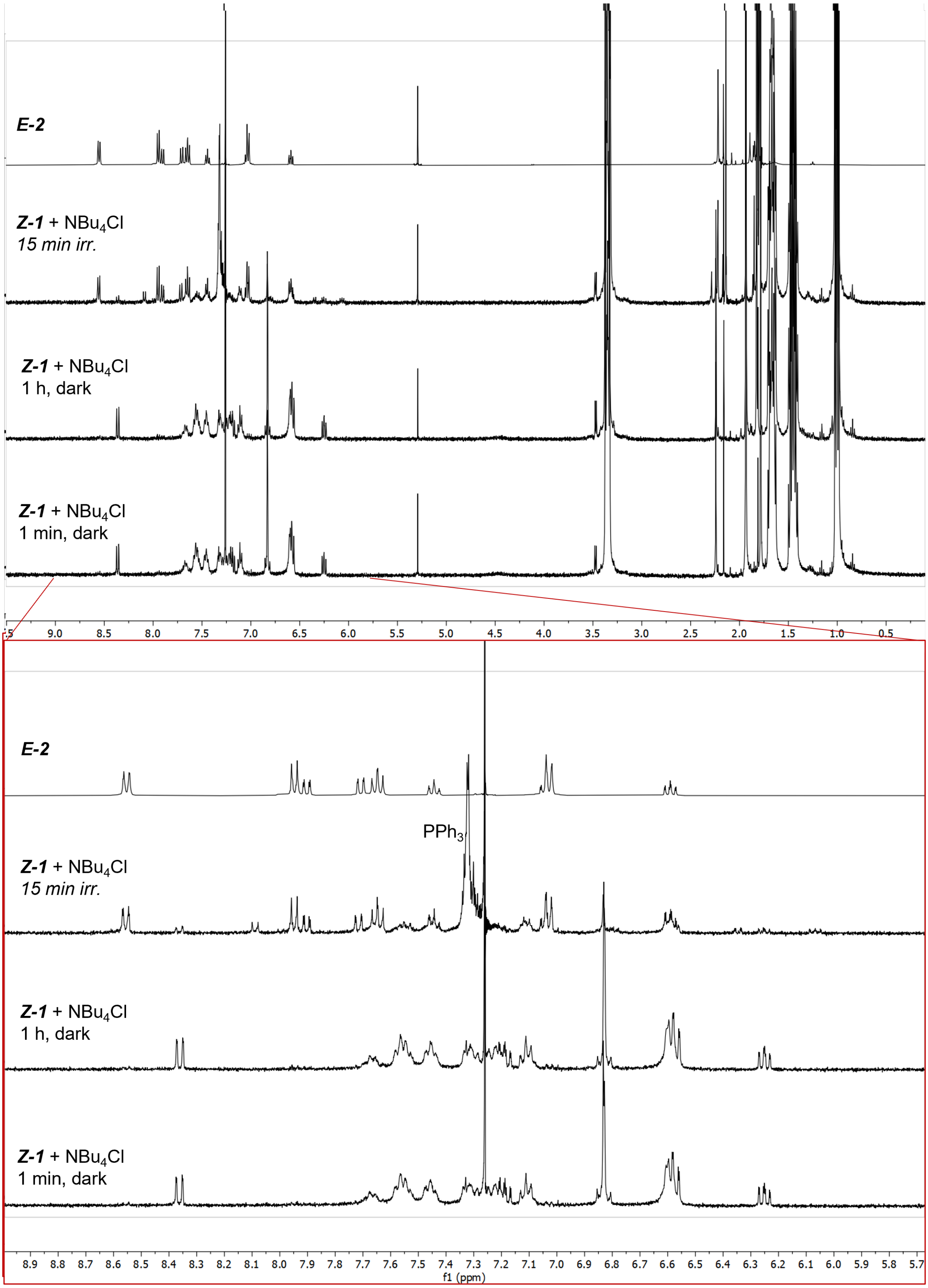
E-2
Z-1 + NBu4Cl
15 min irr.
Z-1 + NBu4Cl
1 h, dark
Z-1 + NBu4Cl
1 min, dark
PPh3
f1 (ppm)

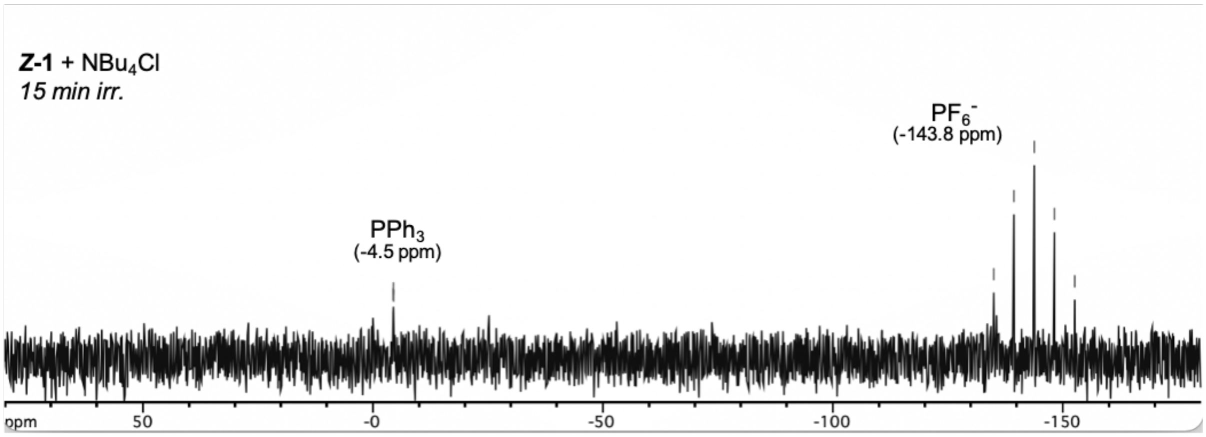

Figure S9. (Top) Evolution of ^{1}H NMR (400 MHz) spectrum of ***Z*-1** (2.8 mM) and tetrabutylammonium chloride NBu_4Cl (5 equiv) in $CDCl_3$ for 1 h in the dark; and 15 min upon white light irradiations (compared with ^{1}H NMR spectrum of ***E*-2**). Inset: Zoom of the aromatic regions. (Bottom) ^{31}P NMR (162 MHz) spectrum of ***Z*-1** (2.8 mM) in $CDCl_3$ after tetrabutylammonium chloride NBu_4Cl (5 equiv.) addition and 15 min white light irradiations.

5. Photo-induced reduction of $PdCl_2$

In an NMR tube, ***Z*-1** (5 mg, 5×10^{-3} mmol) and $PdCl_2$ (1 mg, 5×10^{-3} mmol) were suspended in $CDCl_3$ (0.6 mL) in the dark. NEt_3 (21 µL, 0.15 mmol) was added, the tube was shaken, and the mixture was left for 1 h in the dark. NMR analyses (^{1}H, ^{31}P) revealed the characteristic signals of ***Z*-1**. Then, the tube was irradiated under white light for 20 min. ^{1}H NMR spectrum revealed the formation of ***E*-2** along with remaining traces of ***Z*-1**. ^{31}P NMR showed three signals: two small (+32.3 ppm: traces of ***Z*-1**, +27.5 ppm: $Pd(PPh_3)_4$) and an intense one (+29.9 ppm; $OPPh_3$), without traces of free PPh_3 (-4.8 ppm) or $PdCl_2(PPh_3)_2$ (+24 ppm) (Figure 2). The assignment was performed based on the literature values,[3] and additional experimental data (Scheme S3 and S4). It is worth to remind that the chemical shift strongly depends on the overall concentration of the compound.

Table S2. ^{31}P NMR chemical shifts of phosphorus compounds in $CDCl_3$.

Entry	Compound	Experimental δ_P (PPh_3)	Literature[3] δ_P
1	***Z*-1**	+32.6 ppm	
2	PPh_3	-4.8 ppm	-5.4 ppm
3	$PdCl_2(PPh_3)_2$	+24 ppm	+23.3 ppm
4	$OPPh_3$	+29.7 ppm	+29 ppm
5	$Pd(PPh_3)_2$	+33.7 ppm	+33.0 ppm
6	$Pd(PPh_3)_3$		(+11.9, -5.3)[a] ppm
7	$Pd(PPh_3)_4$	+28.3 ppm	+27.7 ppm

Literature values are reported from spectra referenced to external 85% phosphoric acid (δ_P = 0 ppm) whereas no reference was used in our case. [a] Depending on the concentration.

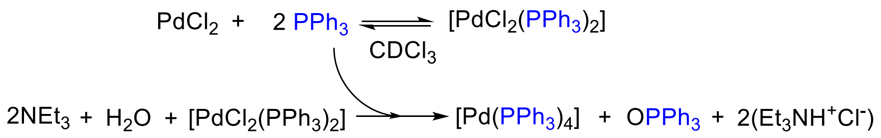

Scheme S3. Reduction of $Pd^{II}Cl_2$ with PPh_3 and NEt_3 in $CDCl_3$.

In an NMR tube, $PdCl_2$ (2 mg; 0.01 mmol) and PPh_3 (6 mg; 0.02 mmol) were suspended in untreated $CDCl_3$ (0.6 mL). ^{31}P NMR spectrum showed PPh_3 signal (-4.8 ppm). After 2 h, the solution became yellow and $[PdCl_2(PPh_3)_2]$ signal (+24.0 ppm) was observed. Then, NEt_3 (21 µL, 0.15 mmol) was added and two new peaks appeared at +29.7 and +28.3 ppm corresponding to the formation of $OPPh_3$ and $[Pd(PPh_3)_4]$ respectively.

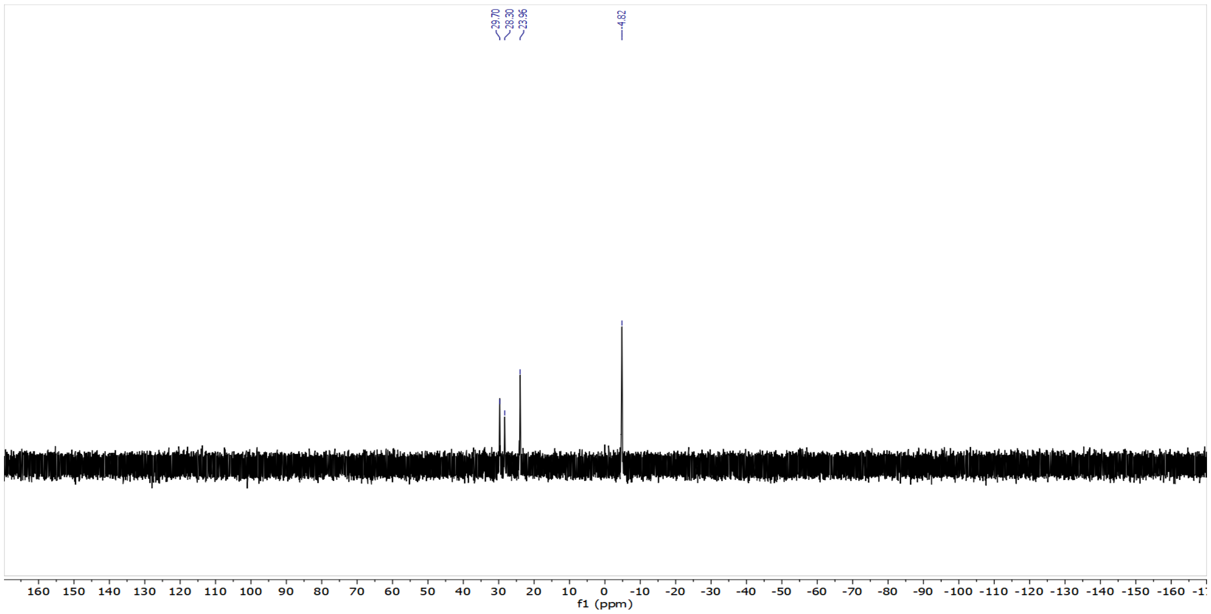

Figure S10. ^{31}P NMR (162 MHz) spectrum of $PdCl_2$ (1 equiv) along with PPh_3 (2 equiv) and after NEt_3 (15 equiv) addition in $CDCl_3$ recorded after 1d.

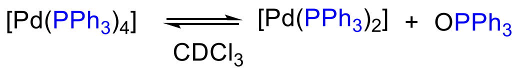

Scheme S4. Dissociation of $[Pd(PPh_3)_4]$ in $CDCl_3$.

When $Pd(PPh_3)_4$ was dissolved in untreated $CDCl_3$, ^{31}P NMR spectrum showed three signals corresponding to the dissociation of the complex: at 33.7 ppm for $Pd(PPh_3)_2$, 29.9 ppm for $OPPh_3$, 28.3 ppm for $Pd(PPh_3)_4$.

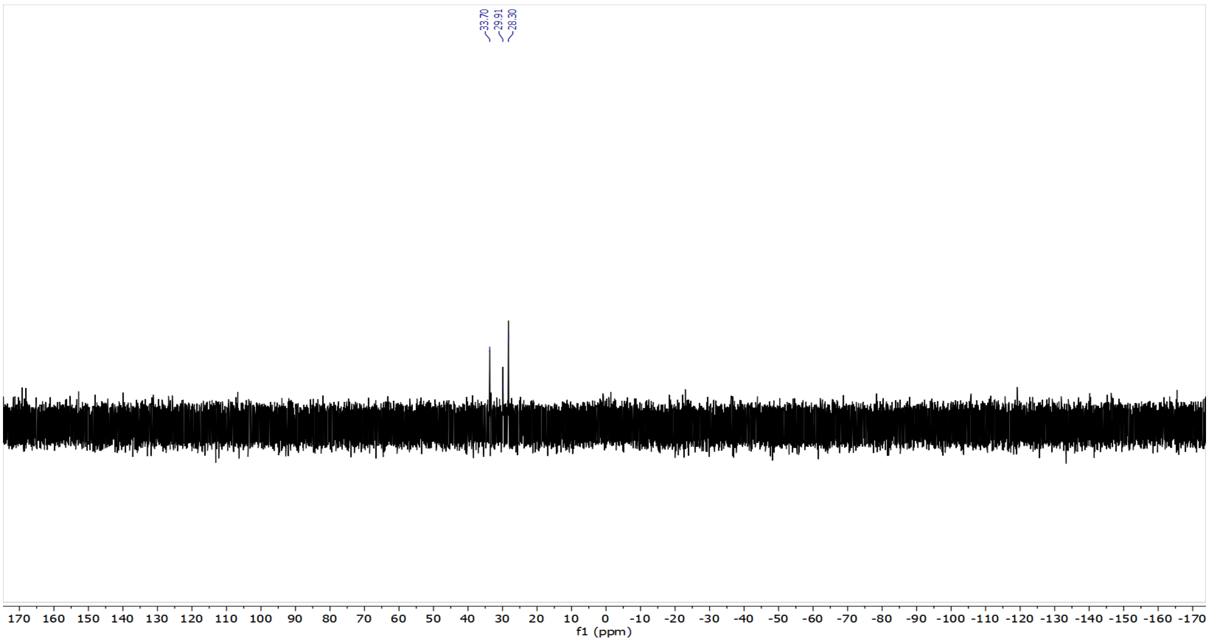

Figure S11. ^{31}P NMR (162 MHz) of $Pd(PPh_3)_4$ dissociation in $CDCl_3$.

6. Sonogashira cross-couplings

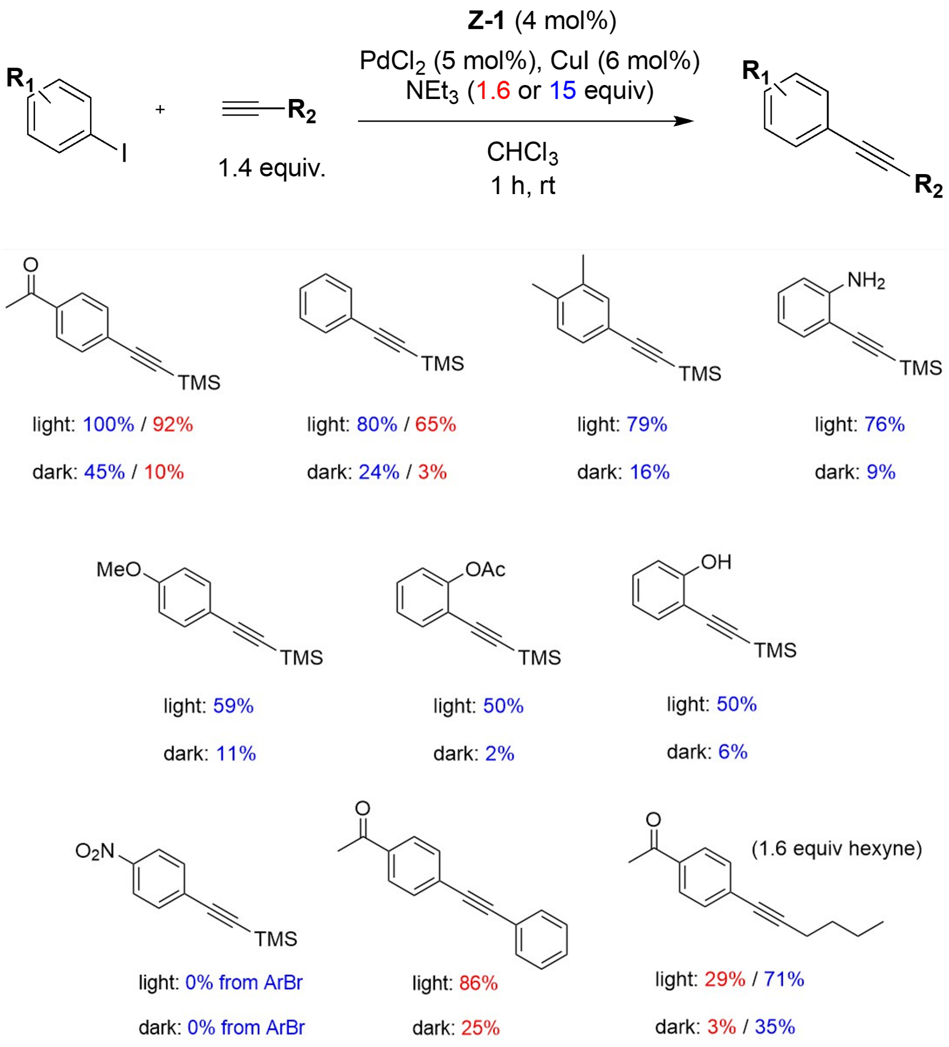

Figure S12. Substrate scope for light-induced Sonogashira coupling using ***Z*-1**.

General procedure: In a dried catalysis tube equipped with a stir bar, $PdCl_2$ (0.5 mg; 2.8×10^{-3} mmol), CuI (0.7 mg; 3.7×10^{-3} mmol), ***Z*-1** (2.5 mg; 2.4×10^{-3} mmol), iodophenyl(R_1) (0.057 mmol) were charged under argon. $CHCl_3$ (1.5 mL) was added, and argon was bubbled into the solution for 1 min. Subsequently, NEt_3 (1.6 equiv.: 13 µL or 15 equiv:125 µL) and acetylene (0.078 mmol or 0.091 mmol for 1-hexyne) were added to the mixture. The reaction mixture was stirred at rt for 1 h in the dark (tube covered with aluminium foal) or upon white light (LED) irradiations using our homemade reactor (Figure S1). Aqueous saturated solution of NH_4Cl was added and the mixture was diluted in CH_2Cl_2. The two phases were separated, and the aqueous phase was extracted three times with CH_2Cl_2. The combined organic phases were dried over $MgSO_4$, filtrated, and concentrated under reduced pressure. The crude residue was analysed by ^1H NMR to determine the conversion yield of the coupling, in agreement with the ^1H NMR data reported in the literature (R_2 = TMS and R_1= Ac,[4] H,[5] 3,4-dimethyl, 2-NH_2,[6] 4-OMe,[7] 2-OAc,[8] 2-OH,[6b]; R_1 = Ac and R_2= Ph,[9] R_2=n-Bu[10]).

R_1=4-Ac; R_2=TMS: ^1H NMR (400 MHz, $CDCl_3$): δ 7.88 (d, *J* = 8.2 Hz, 2H), 7.53 (d, *J* = 8.2 Hz, 2H), 2.59 (s, 3H), 0.27 (s, 9H).

R_1=H; R_2=TMS: ^1H NMR (400 MHz, $CDCl_3$): δ 7.48-7.45 (m, 2H), 7.35-7.28 (m, 3H), 0.25 (s, 9H).

R_1=3,4-dimethyl; R_2=TMS: ^1H NMR (400 MHz, $CDCl_3$): δ 7.24 (s, 1H), 7.19 (d, *J* = 7.2 Hz, 1H), 7.03 (d, *J* = 7.2 Hz, 1H), 2.23 (s, 3H), 2.20 (s, 3H), 0.22 (s, 9H).

R_1=2-NH_2; R_2=TMS: ^1H NMR (400 MHz, $CDCl_3$): δ 7.29 (dd, *J* = 7.7 Hz, *J* = 1.3 Hz, 1H), 7.10 (dt, *J* =7.7 Hz, *J* = 1.4 Hz, 1H), 6.70-6.60 (m, 2H), 4.22 (br, 2H), 0.26 (s, 9H).
R_1=4-OMe; R_2=TMS: ^1H NMR (400 MHz, $CDCl_3$): δ 7.39 (d, *J* = 8.8 Hz, 2H), 6.81 (d, *J* = 8.8 Hz, 2H), 3.80 (s, 3H), 0.23 (s, 9H).

R_1=2-OAc; R_2=TMS: ^1H NMR (400 MHz, $CDCl_3$): δ 7.50 (dd, *J* = 7.6 Hz, *J* = 1.5 Hz, 1H), 7.34 (td, *J* = 7.6 Hz, *J* = 1.5 Hz, 1H), 7.18 (t, *J* = 7.6 Hz, 1H), 7.07 (d, *J* = 7.6 Hz, 1H), 2.33 (s, 3H), 0.25 (s, 9H).

R_1=2-OH; R_2=TMS: ^1H NMR (400 MHz, $CDCl_3$): δ 7.34 (dt, *J* = 7.7 Hz, *J* = 1.7 Hz, 1H), 7.30-7.20 (m, 1H), 6.94 (d, *J* = 8.3 Hz, 1H), 6.85 (t, *J* = 7.5 Hz, 1H), 5.83 (s, OH), 0.28 (s, 9H).

R_1=4-Ac; R_2=Ph: ^1H NMR (300 MHz, $CDCl_3$): δ 7.93 (d, *J* = 8.6 Hz, 2H), 7.60 (d, *J* = 8.6 Hz, 2H), 7.56-7.52 (m, 2H), 7.36-7.33 (m, 3H), 2.60 (s, 3H).

R_1=4-Ac; R_2=n-Bu: ^1H NMR (300 MHz, $CDCl_3$): δ 7.82 (d, *J* = 8.6 Hz, 2H), 7.40 (d, *J* = 8.6 Hz, 2H), 2.53 (s, 3H), 2.38 (t, *J* = 7.0 Hz, 2H), 1.57-1.39 (m, 4H), 0.90 (t, *J* = 7.0 Hz, 3H).

For the kinetic studies (Figure 3), the general procedure was performed in sealed NMR tube under argon, using 4-iodoacetophenone (14 mg; 0.057 mmol) in $CDCl_3$. TMS-acetylene and NEt_3 were added (t=0s) and the tube was vigorously shaken just before recording the first ^1H NMR spectrum. 200 spectra were recorded, one each 39s. The conversion yields were determined based on the relative integrals of the TMS signal for the product with respect to the remaining signals corresponding to the aromatic H of iodoaryl.

7. Appendix

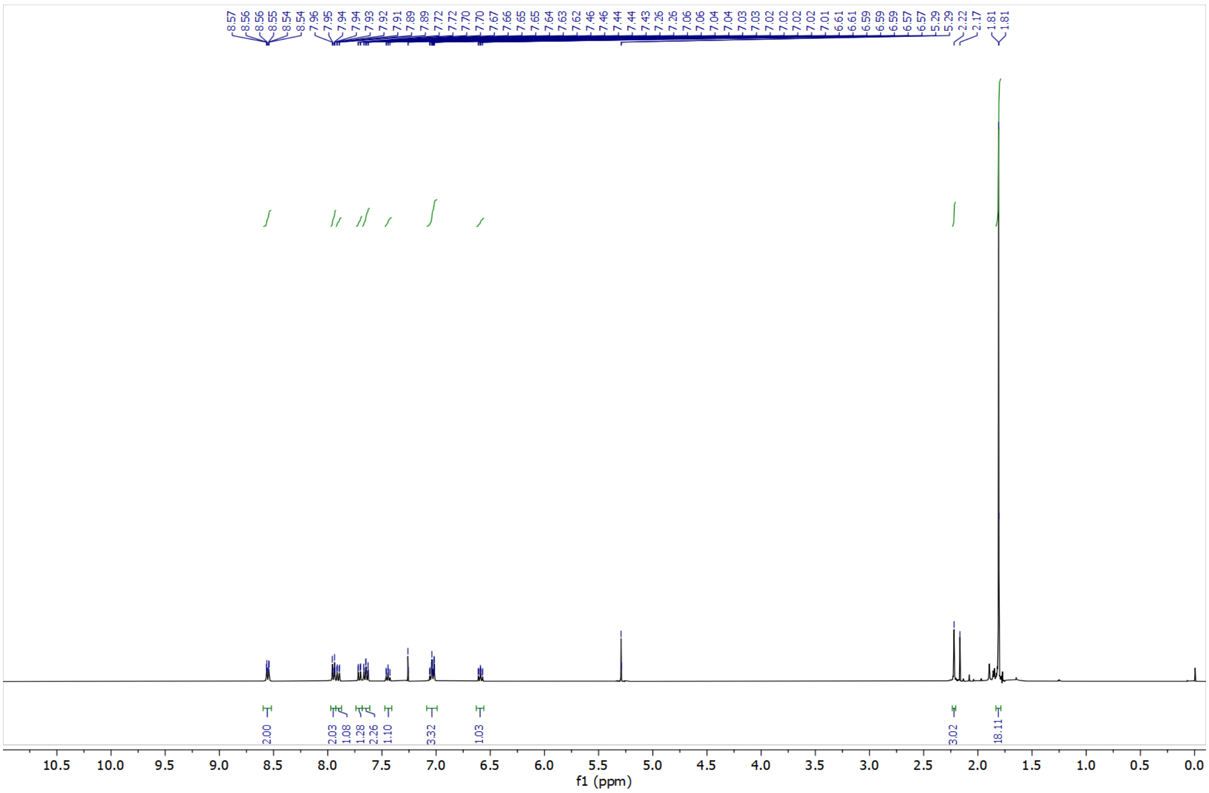

Figure S13: ^{1}H NMR (400 MHz) spectrum of ***E*-2** in $CDCl_3$.

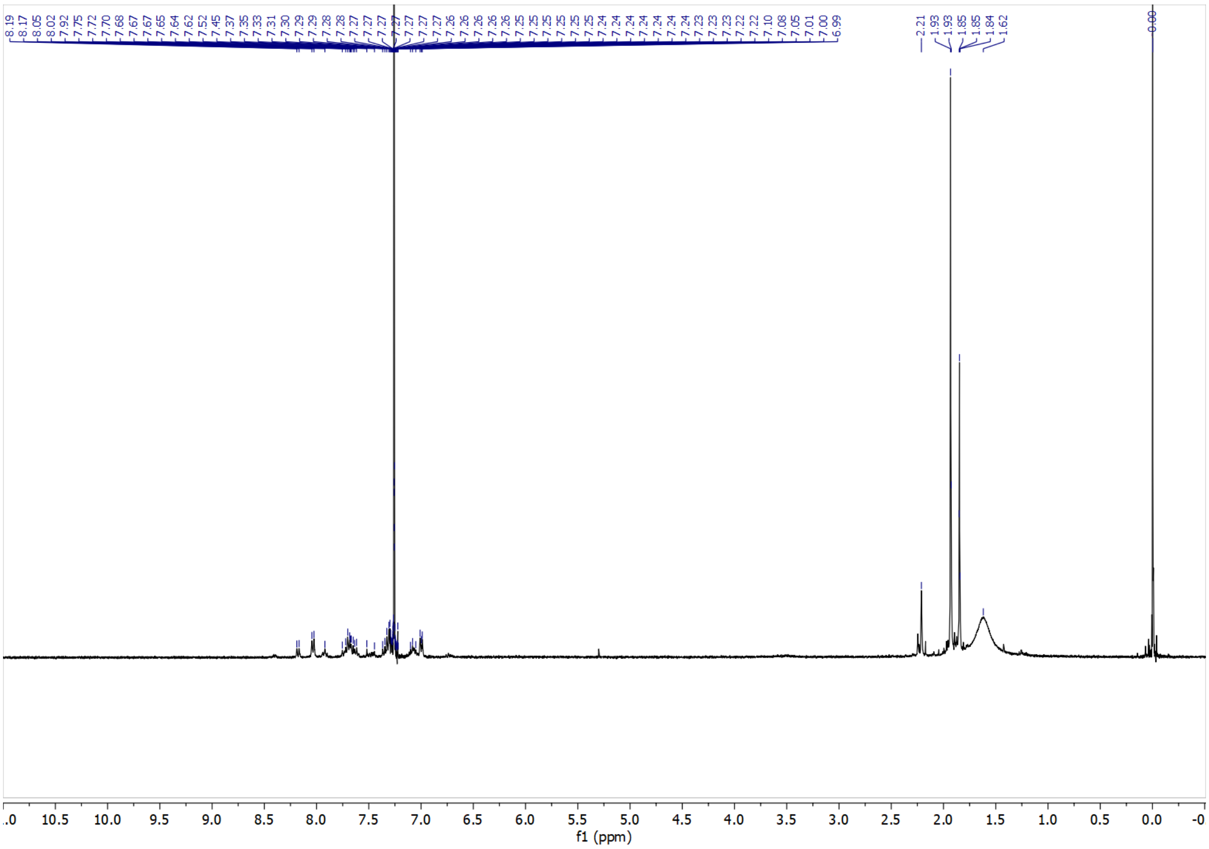

Figure S14. ^{1}H NMR (400 MHz) spectrum of ***E*-S1** in $CDCl_3$. (very low solubility)

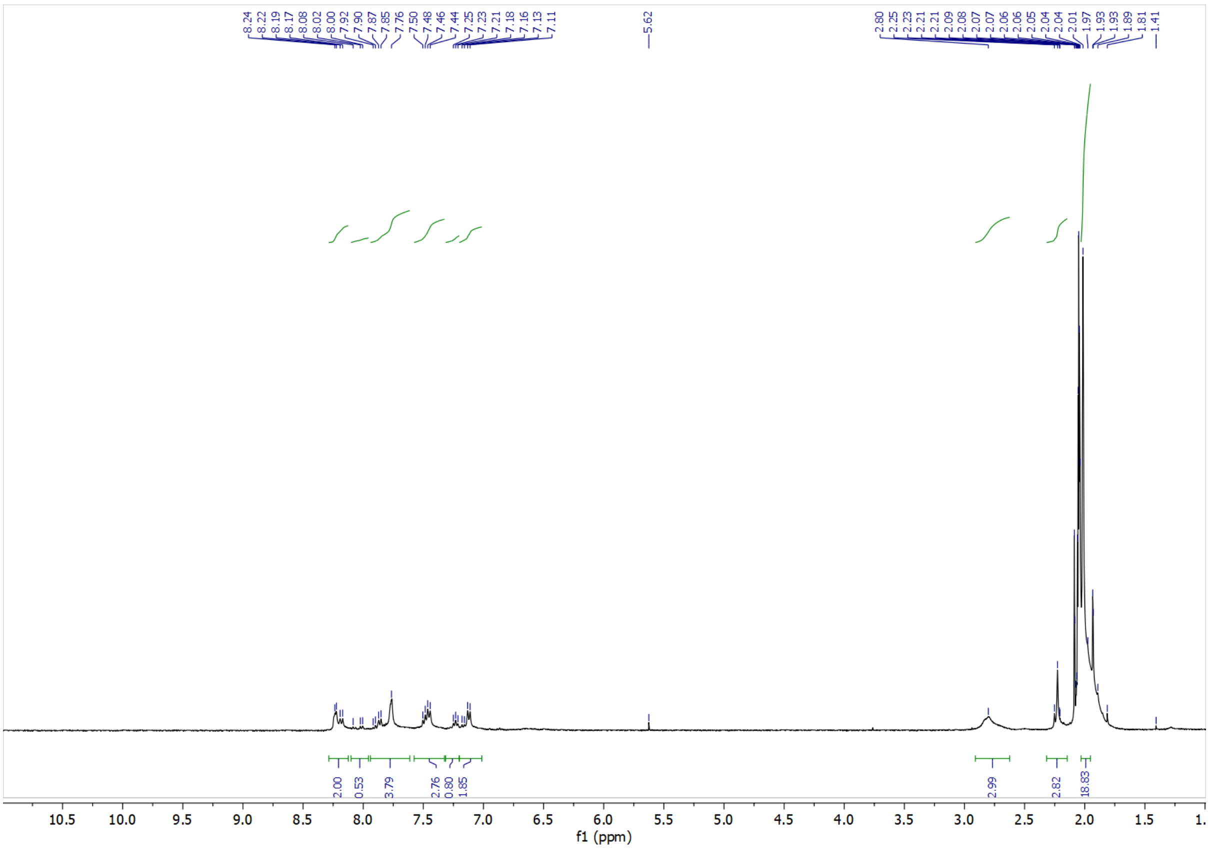

Figure S15. ^{1}H NMR (400 MHz) spectrum of ***E*-S1** in acetone-d_6.

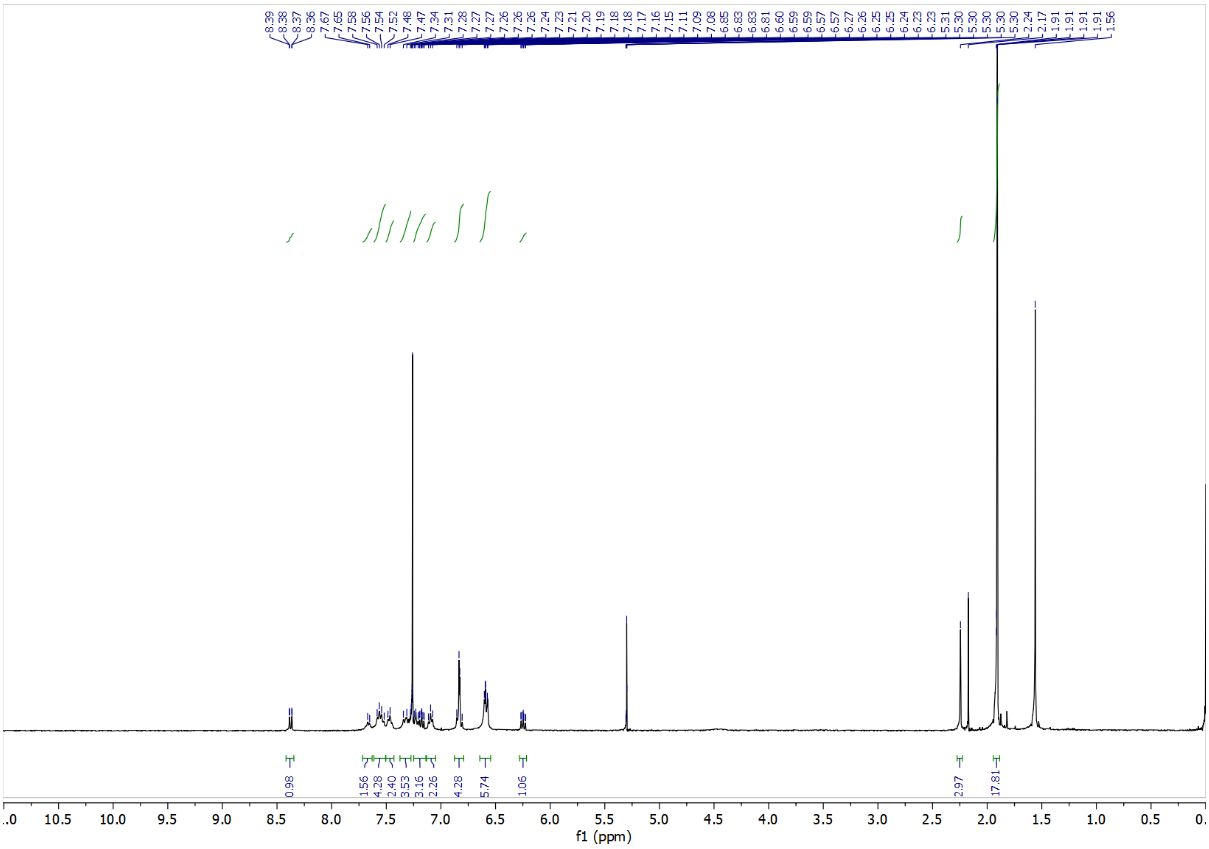

Figure S16. ^{1}H NMR (400 MHz) spectrum of ***Z*-1** in $CDCl_3$. (traces of CH_2Cl_2 and acetone trapped in the compound).

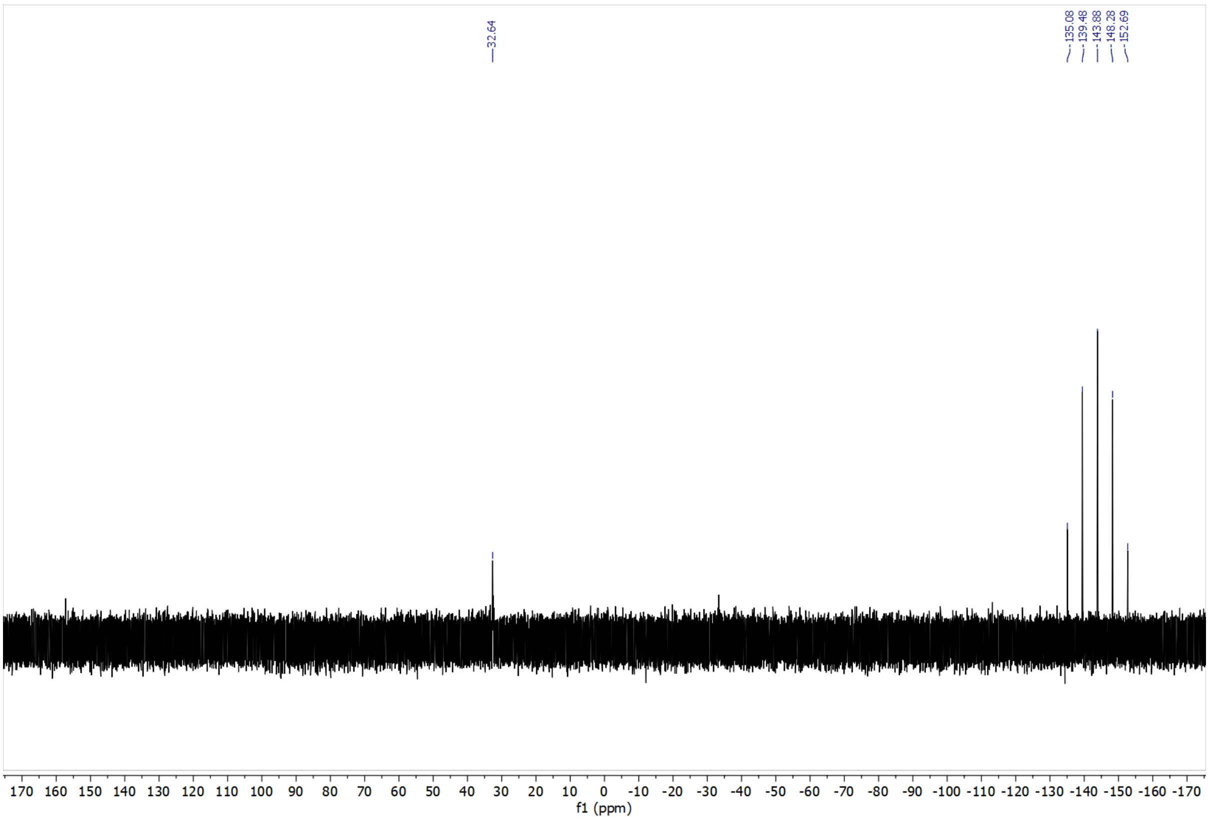

Figure S17. ^{31}P NMR (162 MHz) spectrum of ***Z*-1** in $CDCl_3$.

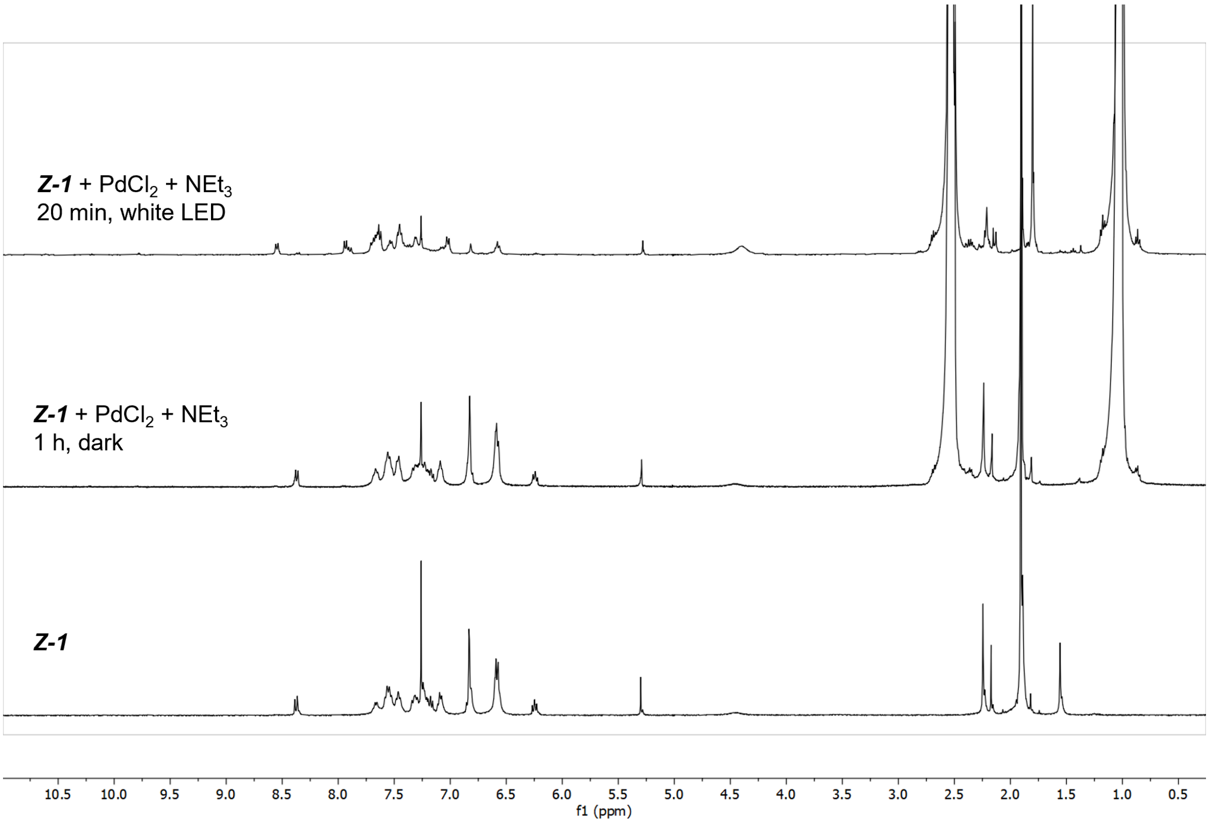

Figure S18. Evolution of ^{1}H NMR (400 MHz) spectrum of Bottom: ***Z*-1** (Bottom); Middle: along with $PdCl_2$ (1 equiv) and NEt_3 (30 equiv) in the dark for 1 h; and Top: after 20 min upon white light irradiations revealing the formation of ***E*-2**. (zoom in main text)

8. References

[1] C. Deo, N. Bogliotti, P. Retailleau, J. Xie, *Organometallics* **2016**, *35*, 2694-2700.

[2] C. Deo, N. Bogliotti, R. Métivier, P. Retailleau, J. Xie, *Organometallics* **2015**, *34*, 5775-5784.

[3] M. Gazvoda, M. Virant, B. Pinter, J. Košmrlj, *Nat. Commun.* **2018**, *9*, 4814.

[4] G. Fabrizi, A. Goggiamani, A. Sferrazza, S. Cacchi, *Angew. Chem. Int. Ed.* **2010**, *49*, 4067-4070.

[5] H. A. Stefani, R. Cella, F. A. Dörr, C. M. P. de Pereira, F. P. Gomes, G. Zeni, *Tetrahedron Lett.* **2005**, *46*, 2001-2003.

[6] a) N. Sakai, K. Annaka, T. Konakahara, *Org. Lett.* **2004**, *6*, 1527-1530; b) R. Álvarez, C. Martínez, Y. Madich, J. G. Denis, J. M. Aurrecoechea, Á. R. de Lera, *Chem. – Eur. J.* **2010**, *16*, 12746-12753.

[7] Y. Araki, K. Kobayashi, M. Yonemoto, Y. Kondo, *Org. Biomol. Chem.* **2011**, *9*, 78-80.

[8] M. Wahab Khan, M. Jahangir Alam, M. A. Rashid, R. Chowdhury, *Bioorg. Med. Chem.* **2005**, *13*, 4796-4805.

[9] A. R. Gholap, K. Venkatesan, R. Pasricha, T. Daniel, R. J. Lahoti, K. V. Srinivasan, *J. Org. Chem.* **2005**, *70*, 4869-4872.

[10] A. Köllhofer, H. Plenio, *Adv. Synth. Catal.* **2005**, *347*, 1295-1300.
